# A computational knowledge-base elucidates the response of *Staphylococcus aureus* to different media types

**DOI:** 10.1371/journal.pcbi.1006644

**Published:** 2019-01-09

**Authors:** Yara Seif, Jonathan M. Monk, Nathan Mih, Hannah Tsunemoto, Saugat Poudel, Cristal Zuniga, Jared Broddrick, Karsten Zengler, Bernhard O. Palsson

**Affiliations:** 1 Department of Bioengineering, University of California, San Diego, La Jolla, CA, United States of America; 2 Division of Biological Sciences, University of California San Diego, La Jolla, CA, United States of America; 3 Department of Pediatrics, University of California, San Diego, La Jolla, CA, United States of America; US Army Medical Research and Materiel Command, UNITED STATES

## Abstract

*S*. *aureus* is classified as a serious threat pathogen and is a priority that guides the discovery and development of new antibiotics. Despite growing knowledge of *S*. *aureus* metabolic capabilities, our understanding of its systems-level responses to different media types remains incomplete. Here, we develop a manually reconstructed genome-scale model (GEM-PRO) of metabolism with 3D protein structures for *S*. *aureus* USA300 str. JE2 containing 854 genes, 1,440 reactions, 1,327 metabolites and 673 3-dimensional protein structures. Computations were in 85% agreement with gene essentiality data from random barcode transposon site sequencing (RB-TnSeq) and 68% agreement with experimental physiological data. Comparisons of computational predictions with experimental observations highlight: 1) cases of non-essential biomass precursors; 2) metabolic genes subject to transcriptional regulation involved in Staphyloxanthin biosynthesis; 3) the essentiality of purine and amino acid biosynthesis in synthetic physiological media; and 4) a switch to aerobic fermentation upon exposure to extracellular glucose elucidated as a result of integrating time-course of quantitative exo-metabolomics data. An up-to-date GEM-PRO thus serves as a knowledge-based platform to elucidate *S*. *aureus’* metabolic response to its environment.

## Author summary

Environmental perturbations (e.g., antibiotic stress, nutrient starvation, oxidative stress) induce systems-level perturbations of bacterial cells that vary depending on the growth environment. The generation of omics data is aimed at capturing a complete view of the organism’s response under different conditions. Genome-scale models (GEMs) of metabolism represent a knowledge-based platform for the contextualization and integration of multi-omic measurements and can serve to offer valuable insights of system-level responses. This work provides the most up to date reconstruction effort integrating recent advances in the knowledge of *S*. *aureus* molecular biology with previous annotations resulting in the first quantitatively and qualitatively validated *S*. *aureus* GEM. GEM guided predictions obtained from model analysis provided insights into the effects of medium composition on metabolic flux distribution and gene essentiality. The model can also serve as a platform to guide network reconstructions for other *Staphylococci* as well as direct hypothesis generation following the integration of omics data sets, including transcriptomics, proteomics, metabolomics, and multi-strain genomic data.

## Introduction

Methicillin-resistant *Staphylococcus aureus* (MRSA) USA300 strains have emerged as the predominant cause of community-associated infections in the United States, Canada, and Europe [1]. Today in the United States more deaths are attributed to MRSA infections than to HIV/AIDS [2,3]. USA300 was first isolated in September, 2000, and has been implicated in wide-ranging and epidemiologically unassociated outbreaks of skin and soft tissue infections in healthy individuals [4]. In 2006, the CDC reported that 64% of MRSA isolated from infected patients were of the USA300 strain type, an increase of 11.3% since 2002 [5], indicating a rapid spread throughout the country. Today, vancomycin resistance amongst *S*. *aureus* strains is on the rise, further complicating antibiotic treatment [6]. USA300 is capable of producing rapidly-progressing, fatal conditions in humans that cause a wide variety of diseases, ranging from superficial skin and soft tissue infections to life-threatening septicaemia, endocarditis, and toxic shock syndrome. Many efforts are geared towards designing new antibiotic regimens to combat MRSA. However, these endeavors are impaired by the lack of replicability in antibiotic potency and bioactivity across different media [7]. Little is known about the systems-level effects of the nutritional environment on *S*. *aureus* growth and metabolism.

While multi-omics data-sets allow for the interrogation of complex interactions occurring on a cellular level, the results can often be hard to interpret. Thus, there is a need for a common knowledge base that enables the integration of disparate data types. Genome-scale models (GEMs) of metabolism have been successfully utilized as a common platform for omics data contextualization and integration [8,9]. GEMs represent mathematically structured knowledge bases of metabolism that contain all of the molecular mechanisms known to occur in an organism. They are built through iterative curation efforts and are constantly updated to reflect the current state of knowledge pertaining to the organism [10]. The *S*. *aureus* GEM has undergone several such iterations over the past 15 years [11–14]. The more recent iterations relied more heavily on semi-automated workflows whereby annotations were pooled from online databases. Unfortunately, online databases rely on a combination of manual curation and sequence homology gene function assignment which is often not organism specific. In general, the more manual curation that goes into a GEM, the more reliable and organism-specific the GEM derived predictions are [15]. The rise of antibiotic resistance amongst *S*. *aureus* strains has created strong momentum in the field of molecular biology and many novel *S*. *aureus-*specific mechanisms have been discovered over the past decade. However, many online databases as well as the current *S*. *aureus* GEM [11] are still lagging behind and do not reflect newly uncovered metabolic capabilities.

In this work, we developed an *S*. *aureus* str. JE2 (strain LAC cured of its plasmids) GEM integrated with protein structures and used a combination of experimental data and computational methods to analyze systems-level metabolic characteristics under different growth conditions [16]. We geared our efforts towards incorporating the newly discovered molecular mechanisms and metabolic pathways of *S*. *aureus* into an updated GEM and brought the most recent *S*. *aureus* GEM through one reconstruction iteration [15]. This iteration is guided by literature findings, experimentally derived gene essentiality data, analysis of protein structures, and microarray growth phenotypes. Such efforts are valuable in that the final *S*. *aureus* G`EM is up to date with online databases, constitutes a blend of the curation efforts of several groups, and quantitatively and qualitatively recapitulates flux and growth phenotypes. We built condition-specific GEMs by integrating time-course quantitative exo-metabolomic datasets and used flux sampling and predicted gene essentiality to compare the metabolic flux state across growth conditions.

## Results

### Expanding the detail and scope of the reconstruction

**Fig 1:**
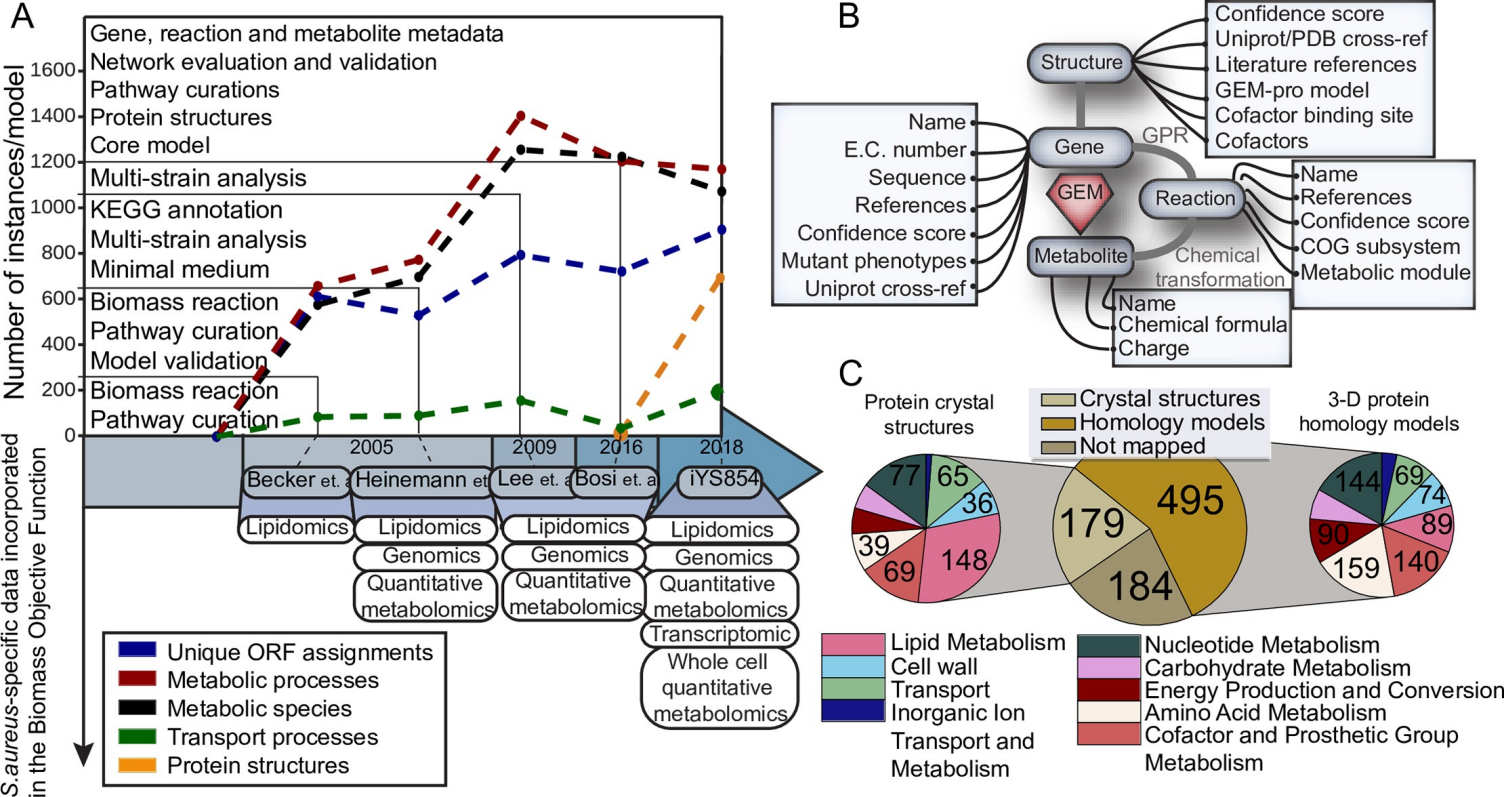
Summary of the reconstruction efforts for *i*YS854. (A) Evolution of *S*. *aureus* genome scale metabolic reconstructions and their biomass objective function from 2005 to 2018. (B) Graphical representation of the four central objects in the *S*. *aureus* GEM; genes, reactions, metabolites, and structures. A representative mapping between all four objects along with relevant metadata are added during the reconstruction process. (C) Percentage of metabolic genes mapped to protein crystal structures and protein homology models, and distribution of metabolic subsystems per category (more details are shown in **Table S4 in S2 Appendix**).

We followed an established workflow for the reconstruction of genome-scale metabolic networks [15] to curate and update the most recent genome-scale model (GEM) of *S*. *aureus [11]* with new content. The basic steps outlined in a reconstruction workflow include: Step 1: building a draft reconstruction from a genome annotation; Step 2: refining the reconstruction using literature evidence; Step 3: converting the reconstruction into a computable format; and Step 4: evaluating and validating the network against experimentally observed phenotypes [15]. We conducted detailed and extensive manual curation that brought about major modifications to the *S*. *aureus* metabolic network across 56 metabolic sub-modules (**Table S1 in S2 Appendix**). Our efforts were guided by a combination of literature review and network evaluation and proceeded in an iterative fashion. *i*YS854 contains 854 unique ORF assignments, 1,202 metabolic processes (excluding biomass and exchange reactions), 1,084 metabolic species, and 681 3D protein structures (**Fig 1A, S1 Data**). We also designed an updated condition-specific biomass objective function “BIOMASS_iYS_wild_type” and a general biomass objective function “BIOMASS_iYS_reduced” (**Fig 1B**). We enriched the objects included in the reconstruction (genes, proteins, reactions, and metabolites) with layers of metadata and cross-references (**Fig 1C**).

#### An updated core *S*. *aureus* model recapitulates realistic flux states

We used the GEM published by Bosi et. all as the starting draft model. However, instead of taking the full reconstruction through one iterative reconstruction step, we initially constrained our efforts to pathways of central metabolism. We built a core *S*. *aureus* metabolic network (iYS103) by selecting metabolic processes across the pentose phosphate pathway (PPP), glycolysis and gluconeogenesis, respiration, the Krebs cycle, glutamine biosynthesis, and transport and exchanges **(S2 Data)**. A core model is useful for applications such as kinetic modeling (where a smaller number of variables may be a useful attribute for computational simulations), educational purposes and analyzing the applicability of new constraint-based algorithms. We observed that *S*. *aureus*’ core metabolism is distinguished by: 1) the presence of a malate:quinone oxidoreductase, lactate:quinone oxidoreductase, and NADH:quinone oxidoreductase; 2) the ability to utilize both oxygen and nitric oxide as electron donors; and 3) the absence of a glyoxylate shunt [17], ubiquinone biosynthesis, and vitamin K biosynthesis, which we removed from the starting model [18]. Surprisingly, some elements of the *S*. *aureus* respiratory pathways remain unknown today or have had their underlying molecular mechanisms uncovered only recently [19–22]. For example, it was recently shown that *S*. *aureus* synthesizes a type 2 non-electrogenic NADH:quinone oxidoreductase [20] that could be coupled indirectly with a three protein complex (*mpsABC*). The latter complex was shown to function for both the generation of membrane potential (*Δψ*) and sodium transport [21]. Manual curation efforts led to the removal of redundant content which had initially allowed for the existence of erroneous energy generating cycles (EGCs). An EGC consists of a set of reactions that together allows for thermodynamically impossible fluxes. When they occur, the model can simulate the free production of energetic cofactors such as ATP and NADP. Such cycles are common when a model has not undergone sufficient curation and validation [23]. While the starting model was capable of producing 13 energy carriers with no nutrient exchange, the final (updated) model could generate none **(see** **Methods**).

#### A module-by-module reconstruction highlights areas of metabolism that have been recently characterized

**Fig 2:**
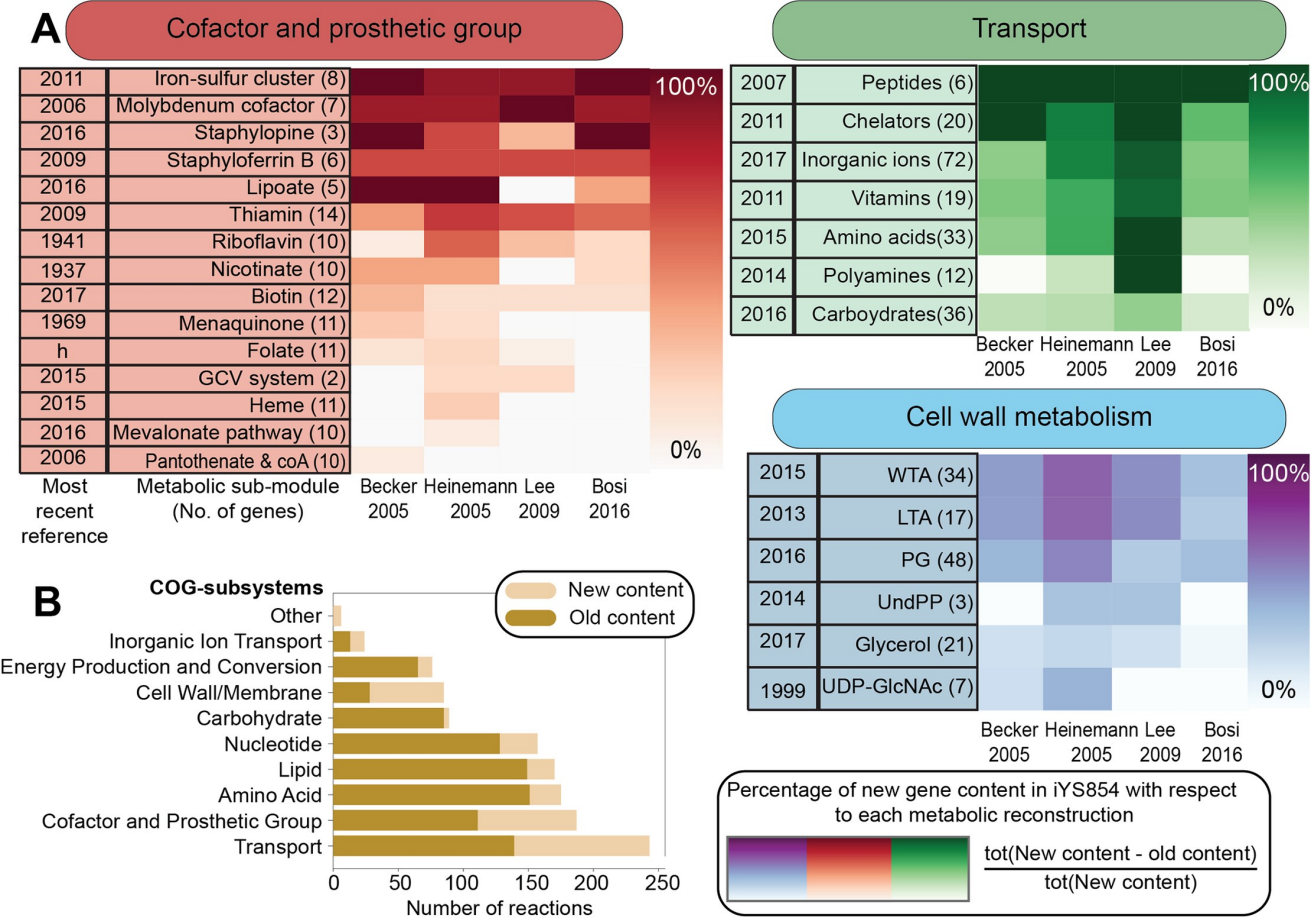
Break down of the novel content in *i*YS854 by metabolic sub-module and COG category. (A) We compared the gene content in *i*YS854 to that of the four previous GEMs of *S*. *aureus* and categorized them by their metabolic sub-modules. For purposes of clarity we only show a subset of the sub-modules across three COG categories: cofactor and prosthetic group metabolism, transport, and cell wall metabolism. The color scale represents the percentage of novel genes in *i*YS864 with respect to previous GEMs (columns) by each metabolic sub-module (rows). We highlight the date for the most recent reference that was added in *i*YS854 for each metabolic sub-module (see S3 Table in S2 Appendix for more details). Genes may have different annotations in previous reconstructions (for example the staphylopine biosynthesis pathway was only uncovered in 2016). Note that “h” represents a metabolic sub-module that was added based on gene homology. (B) We compared the most recently published GEM with *i*YS854 and highlighted the new additions in reaction content per COG module.

Once the core metabolic model was curated, we proceeded to add and curate metabolic modules one at a time to yield the full metabolic network (iYS854). For each module, confidence scores, references, and subsystem annotations were assigned when content was added or modified **(see** **Methods**). We examined more than 50 metabolic sub-modules and added a total of 204 confidence scores and 323 references. The cofactor and prosthetic group metabolic subsystem was expanded the most due to several discoveries spanning *S*. *aureus*-specific metal chelators and metal acquisition systems [24–30] (e.g., Staphylopine [31], Staphyloferrin A [32], and Staphyloferrin B [33]**; Fig 2A and 2B)**. These are relevant because *S*. *aureus* virulence, respiration, and antibiotic resistance have been documented to be dependent on metals [34,35]. A total of 57, 24, and 8 new reactions were added across cell wall metabolism [36–38], amino acid metabolism [39–41], and redox metabolism, respectively [42–44] (**S1 Table in S2 Appendix**). Only 67% of the reactions in the starting reconstruction were assigned to 31 subsystems. We assigned a subsystem (following the Clusters of Orthologous Groups (COGs) classification schema) to all reactions and metabolic module names to 87% of the metabolic reactions. As a result of our reconstruction efforts, we added 214 new ORF assignments, 569 new metabolic processes and, 207 new metabolites and removed 41 ORF assignments, 634 metabolic processes, and 253 metabolites (**Table 1, see** **Methods**). A total of 64 orphan reactions (i.e., reactions with no ORF assignment) were either removed or updated with a gene protein reaction rule.

**Table 1:** Summary of the modifications made to the starting model. A single instance is counted towards a metabolite even when it appears in two different subsystems.

	Metabolic processes	Unique ORF assignments	Unique metabolites
**Changed instance(s)**	110	283	0
**Final reconstruction**	1440	854	1094
**New instances**	566	214	209
**Removed instance(s)**	637	41	253
**Starting reconstruction**	1511	691	1138
**Unchanged instance(s)**	764	367	0

#### The added 3D protein structures span the majority of the reactome

Genome-scale metabolic models have recently gained an additional dimension: 3-dimensional protein structures, in which known metabolic transformations are linked to the 3D structure of the corresponding catalyzing enzyme. In addition to aiding the reconstruction process by enriching the protein object with details on its molecular mechanism as well as its 3 dimensional geometry, structural systems biology has implications in drug development and personalized medicine [45], and enables the analysis of structural features at the network level [46,47]. We used a standardized workflow [48] to search the protein data bank (PDB) [49] for matching content and conduct homology modelling **(see** **Methods**). Overall, 401 genes of the USA300 str. JE2 genome were found to have a close match, of which 183 were mapped to a total of 501 metabolic processes **(Fig 1C)**. A total of 30% of the protein structures were mapped to lipid metabolism and 15% were mapped to nucleotide metabolism. We used the cross-referenced publications for each of the modelled protein structures to validate and further guide our reconstruction efforts. The remaining 686 modelled genes that were not mapped to an experimentally crystallized protein structure required homology modelling, of which we have modeled 65.4% (449 non-transport related proteins). In total, 79% of the genes included in this reconstruction were mapped to a protein structure.

#### An updated biomass function

Once the networks were built and curated, we proceeded to step 3 of the reconstruction workflow and designed an updated biomass objective function. Such a function represents bacterial growth through the drain of biomass precursors, and directly influences the computed activity level across metabolic submodules [10,50]. The choice of such precursors and their respective rate of drain (or biomass coefficient) varies between conditions and is specific to the organism of interest. The advent of higher resolution metabolomics and other omics datasets (e.g. genomics, transcriptomics) represents a major advance for the design of the biomass objective function [51].

The biomass objective function for the starting model was adapted from a combination of the biomass function designed for *Bacillus subtilis* (iYO844), *E*. *coli*, and *S*. *aureus* specific lipidomic data. With the purpose of excluding non *S*. *aureus* specific content, we adapted the ratios for the macromolecular composition of *S*. *aureus* reported in Heinemann et. al [12]. A combination of *S*. *aureus* omics data measurements was then used to adjust the choice of biomass precursors and their rate of drain (including genomic, transcriptomic, and intracellular quantitative metabolomic data; **Fig 1A, see** **Methods**). The content and coefficients for the pool of solutes were obtained from intracellular quantitative metabolomics measurements for *S*. *aureus* cultures in a chemically defined medium (CDMG) [52]. However, bacterial cells produce metabolic intermediates that can vary dramatically between growth conditions rendering the measured pool of solutes the most dynamic category of precursors in the biomass objective function. Therefore, it should be left out of simulations when growth on other media types is modelled. We thus designed a second biomass objective function (termed “BIOMASS_iYS_reduced”) which can be used when the culture medium is not CDMG. Finally, trace metals were added as a result of inspecting the metal cofactors annotated as essential for the activity of proteins in the GEM-PRO model.

### Experimental validation of the model

We proceeded to validate the GEM against experimental observations (step 4). In this step of the reconstruction, analyzing the discrepancies between model predictions and experimental outcomes can highlight model errors and areas of knowledge gaps. Ultimately, the systems-level view can give a deeper understanding of the organism’s metabolism and guide the generation of testable hypotheses.

#### *i*YS854 suggests metal cofactor promiscuity in *S*. *aureus*

We set out to verify that the GEM can successfully recapitulate some of the known growth phenotypes of *S*. *aureus*. We simulated growth *in silico* on seven chemically defined media types; five of which can support growth of *S*. *aureus* strains: 1) synthetic nasal extract (SNM3) [53]; 2) Chemically Defined Media (CDM): CDM [39]; 3) CDMG (CDM+glucose) [39,54]; 4) CDMgal (CDM+galactose) [55]; and 5) CDMG2 (CDM.v2+glucose) [56], and two of which cannot: 1) glucose+M9 minimal medium and 2) RPMI. RPMI and SNM3 are synthetic physiological media for plasma and the nose, respectively. We found that growth could be successfully simulated *in silico* on CDM, CDMG, and CDMgal, but that iron supplementation was required for growth on SNM3 and supplementation with zinc and molybdate was required for CDMG2. Trace metals are known to play an important role in protein function and stability as well as in redox maintenance in *S*. *aureus* [57–60]. Whether *S*. *aureus* can survive without one of these trace metals remains to be determined. Growth on RPMI or M9+glucose minimal medium was unsuccessful *in silico*. The model predicted that supplementation with manganese, zinc, and molybdate was required for RPMI, while supplementation with niacin and thiamin was required for glucose+M9 minimal medium. Interestingly, *S*. *aureus* strains have been shown to exhibit both niacin and thiamin auxotrophies [61] as a result of the absence of tyrosine lyase and nicotinate-nucleotide diphosphorylase. *S*. *aureus* can grow under both aerobic and anaerobic conditions and utilize nitrate as an alternate electron acceptor. When anaerobic conditions were simulated, the model predicted a 52% decrease in biomass yield with respect to aerobic conditions. The addition of nitrate to the simulated anaerobic minimal medium yielded a 70% increase in biomass yield (see **Table 2, see** **Methods**). Similar simulations run on the starting model showed no difference in predicted growth rate between aerobic, anaerobic and anaerobic + nitrate conditions. Additionally, supplementation with both purine and L-leucine was required across several media types.

**Table 2:** Results of growth simulations for *i*YS864 on seven defined media.

Simulated medium	Observed Growth *in vivo*	Simulated Growth *in vitro (1/hr)*	Proposed supplementations	Growth upon supplementation	Anaerobic Growth upon supplementation	Anaerobic Growth upon supplementation in the presence of nitrate
**CDM**	1	1.92475	None	1.92475	0.115168	0.858722
**CDMgal**	1	2.92622	None	2.92622	1.24279	1.90119
**CDMG**	1	3.02115	None	3.02115	1.2914	1.96277
**CDMG2**	1	0	Zinc AND Molybdate	3.58009	1.59875	3.03455
**Glucose+M9 minimal medium**	0	0	Thiamin AND Nicotinamide	1.3081	0.621565	1.21695
**RPMI**	0	0	Mn2+ AND Zinc AND Fe2+ AND Molybdate	3.74914	1.59E-15	-3.54E-14
**SNM3**	1	0	Fe2+	3.10515	1.3839	2.62512

#### *i*YS854 has an expanded range of catabolic capabilities

To estimate the accuracy of the model’s carbon catabolism capability, we experimentally tested for the ability of strain USA300-TCH1516 to catabolize 69 carbon sources using a high-throughput BIOLOG phenotypic array **(Table S7 in S2 Appendix)**. A total of 53 carbon sources supported *in vitro* growth and we obtained a 68.3% agreement with *in silico* predictions (see **Methods**). The comparison exposed eleven false positives and ten false negatives.

False positives occur when the model predicts successful growth when none is observed experimentally. They may result from additional constraints which are not accounted for in the model such as regulatory and kinetic constraints [62]. For example, L-arginine and L-proline fell under the category of false positives and the genes involved in the biosynthesis of L-glutamate from both metabolites are subject to carbon catabolite repression and CcpA [39]. False positives also included adenosine, D-alanine, fumarate, L-aspartyl-glycine, L-alanyl-glycine, L-malate, L-threonine, N-acetylneuraminate, and uridine.

False negatives occur when the model predicts no growth on a medium condition when growth is observed experimentally. The carbon sources that fell in this category included 2-oxobutanoate, acetamide, acetate, butyryl-ACP, formate, glycolate, hypoxanthine, L-lysine, L-methionine, and myo-inositol. Interestingly, *S*. *aureus* cannot utilize C2 compounds (such as acetate, glycolate, and formate) as a sole carbon source *in silico* because it lacks the essential reactions present in the reconstruction of other organisms (such as the glyoxylate shunt and pyruvate synthase). However, the model can simulate the assimilation of C2 compounds when L-glutamate uptake is allowed. While the starting model contains exchanges for 77 carbon sources (and predicts 36.4% of growth profiles correctly), only 31 were linked to the rest of the network, for which 64.5% of growth predictions agreed with experimental observations (see **Table S7 in S2 Appendix**).

#### *i*YS854 gene essentiality predictions agree with experimental outcomes

**Fig 3:**
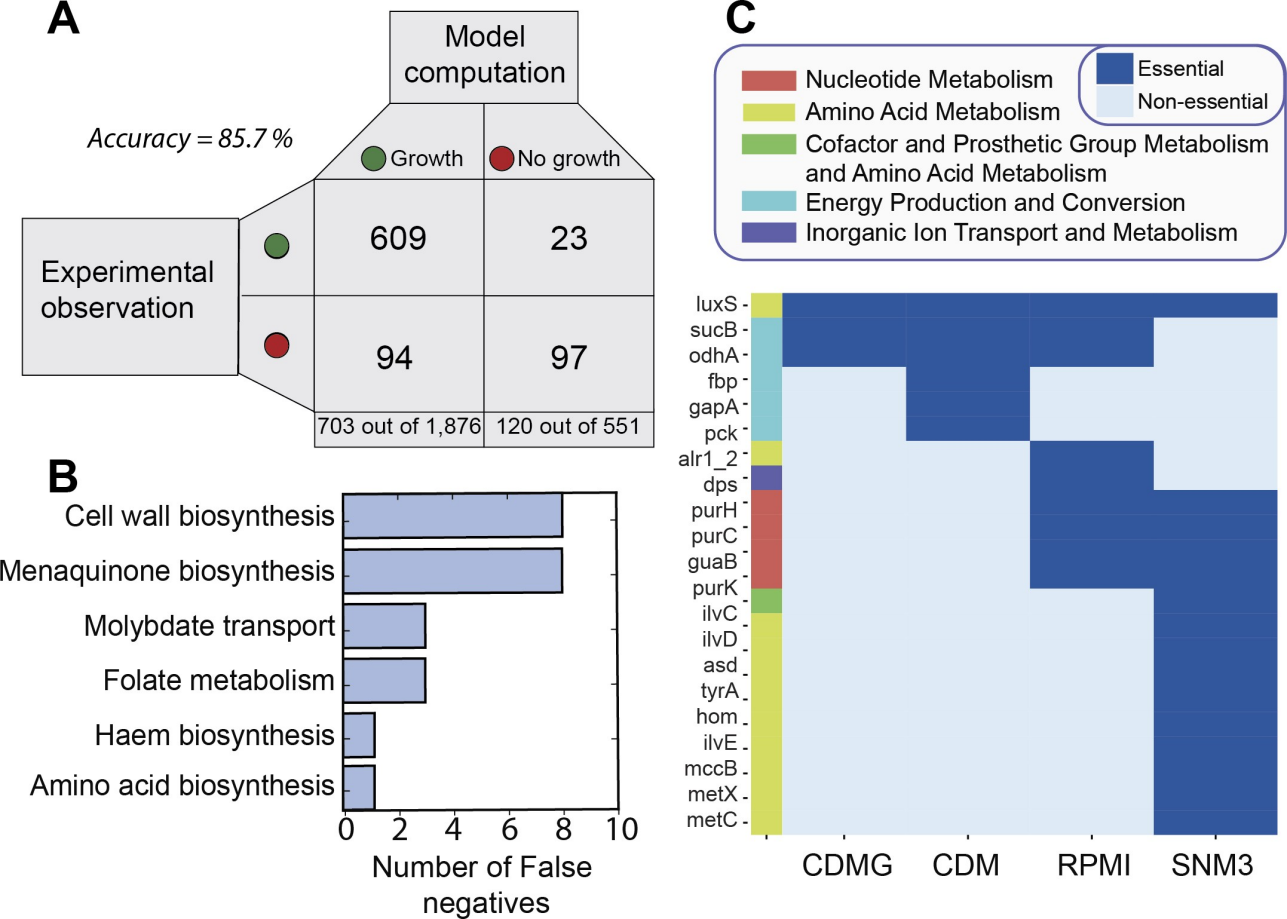
Comparison of *in vivo* vs. *in silico* gene essentiality. (A) Contingency matrix for the comparison of *in silico* gene essentiality predictions of *i*YS854 on rich medium with *in vitro* observations of tn-seq mutants on TSB. The accuracy is 85.7%, which represents an increase of 10.1% with respect to the most recent model [11]. (B) The genes that fell into the category of false negatives were grouped by the biomass precursor in whose biosynthesis they participate. (C) Predictions of gene knockout on growth phenotype across defined media types. Here, we show a subset of four media types and the subset of conditionally essential genes that are not essential in at least one media type. The full data is available in S3 Table and the full cluster map is available in **S1** Appendix.

The robustness of the network against genetic perturbation can be assessed and validated against *in vitro* gene essentiality. Fey et. al recently generated a sequence defined transposon mutant library for 1,952 strains of *S*. *aureus* USA300 str. JE2 [16]. With this method, they identified 579 essential genes for growth on Tryptic Soy Broth (TSB). We simulated the effect of 854 single gene knock-outs on biomass production in rich medium *in silico* and found 121 essential genes **(see** **Methods),** which amounts to 85.7% agreement with experimental observations (**Fig 3A, S1 Table in S2 Appendix**). The same simulations run with the starting model (which contained a lower number of ORF assignments) yielded only 75.6% agreement in essentiality observations for only 656 genes (**Fig S1 in S1 Appendix**). Analysis of the discrepancies between predictions and observations revealed gaps of knowledge in L-methionine biosynthesis (**S1 Appendix)** and highlighted cases of non-essential protein complex subunits for complexes involved in respiration, glycerol degradation, molybdate uptake, and tryptophan biosynthesis (**S1 Appendix**). We also distinguished true from false isozymes by using a combination of sequence homology and structure homology (obtained from the GEM-PRO) coupled with gene essentiality observations (**S1 Appendix**).

#### *S*. *aureus* can exhibit altered phenotypes

False negatives highlighted cases of non-essential biomass components in S. aureus. A total of 23 modelled gene knockouts were falsely predicted to be lethal upon disruption, including genes that were ‘essential’ for their role in cell wall biosynthesis, menaquinone biosynthesis, molybdate transport folate metabolism, haem biosynthesis, and amino acid biosynthesis (**Fig 3B**). Of the 23 false negatives, eight genes were involved in cell wall metabolism: *tarBS—*wall teichoic acid biosynthesis, *ltaA*, *ugtP*, *mprF*, and *pgpB—*lipoteichoic acid biosynthesis and charging, *bacA*—undecaprenyl phosphate biosynthesis, and *gtaB* UDP-galactose biosynthesis **(Fig S2 in S1 Appendix)**. Both wall teichoic acids and lipoteichoic acids are conditionally dispensable for viability of *S*. *aureus* strains [63,64]. Since we initially included these two components in the biomass objective function, their complete biosynthesis was rendered essential for successful growth *in silico*. Thus, in addition to the measured intracellular pool of solutes, we also propose that the inclusion of WTA and LTA in the biomass objective function is conditional. As such, we adjusted the generalized biomass objective function (‘BIOMASS_iYS_reduced’) to exclude these two precursors.

False negatives also highlight the capability of S. aureus to exhibit the small colony variant phenotype and included several genes involved in: 1) menaquinone biosynthesis; *aroB*, *aroC*, *aroD* and *aroF*, 2) the shikimate pathway, *menF* and *menD*, 3) the mevalonate pathway, and; 4) the isoprenoid pathway—*ispA* and *hepT*. Incidentally, *menD* mutants exhibit the clinical small colony variant phenotype (SCV), which is characterized by slow growth, intracellular persistence, nutrient auxotrophies and altered metabolism [65–67]. *MenD* mutants are auxotrophic for hemin, menadione, and thymidine [68]. Hemin and thymidine but not menadione transport and utilization are included in the reconstruction because there are no known metabolic routes linking menadione to menaquinone-7 in *S*. *aureus*. However, menadione can serve as a precursor for menaquinone-7 in *S*. *aureus* auxotrophs [69]. When we added a temporary menaquinone-7 transport reaction (in addition to allowing for the uptake of hemin and thymidine), we found that *menD* mutant growth was rescued *in silico*. In both *S*. *aureus* and *E*. *coli*, the shikimate pathway is the sole metabolic route known to yield chorismate—which is an essential precursor for the biosynthesis of menaquinone-7, folate intermediates, thiamin, and aromatic amino acids. *AroB E*. *coli* mutants were also found to grow successfully in Luria-Bertani (LB) broth (a rich medium) but not in any of the carbon source + M9 minimal medium combinations [70]. We hypothesize that all of the necessary nutrients essential for growth of the *E*. *coli* and *S*. *aureus aroB* mutants are present in LB and TSB, respectively.

#### *i*YS854 recapitulates mutant phenotypes and uncovers genes subject to regulation involved in Staphyloxanthin production

In addition to identifying essential genes, Fey et. al screened their mutants for pigmentation and mannitol fermentation. They identified seven mutants with reduced mannitol fermentation capability, six of which had transposon insertions within unique ORFs. *i*YS854 correctly predicted that the six genes are essential for mannitol fermentation **(see** **Methods)**. Fey et, al also found a total of 39 mutants to be affected in their pigmentation capability. Of the 39 ORFs containing a transposon insertion, 15 are accounted for their metabolic activity in the reconstruction. Staphyloxanthin is an orange-red carotenoid and its biosynthetic pathway is included in *i*YS854. We proceeded to assess the effect of gene knock-outs on the production of Staphyloxanthin. *i*YS854 predicted that 35 genes completely abrogated Staphyloxanthin production when knocked out *in silico*, while 14 single gene knock-outs almost halved it (including *qoxA*, *qoxB*, *qoxC*, *qoxD*, *cydA*, *cydB*, *narT*, *narX*, *narW*, *narH*, *narG*, *nasD*, *nasE*, and *ctaM*; **see** **Methods, S9 Table in S2 Appendix)**. Of those 35 genes, seven were observed to affect *S*. *aureus*’ pigmentation capability by Fey et. al (including *crtO*, *crtP*, *crtQ*, *crtM*, *crtN* and *ispA*). On the other hand, six of the 15 single gene knock-outs simulated an unchanged pigment yield. The cognate genes are involved in glycolysis (*pdhE1* and *fbp*) and purine biosynthesis (*purA*, *purB*, *gapA*, and *yjbK*). This discrepancy could be due to context-specific transcriptional regulation. Indeed, in another study, *purA S*. *aureus* mutants showed enhanced pigmentation potentially mediated by the enhanced expression of *sigB* [71].

#### Amino acid and purine biosynthesis are essential in synthetic physiological media

We proceeded to assess conditional gene essentiality for each of the simulated media types (including SNM3, CDM, CDMG, CDMgal, CDMG2, M9+glucose, and RPMI). We found that 92 genes were predicted to be essential for growth on all seven media types but not TSB, with 28 genes predicted to be essential in at least one but not all media types (**Fig 3C, Fig S3 in S1 Appendix**). Of the 92 genes, 23 were wrongly predicted to be essential for growth in TSB. The remaining 67 genes were categorized as conditionally essential genes, meaning that the corresponding mutants can grow in the medium of interest when supplemented with the right combination of nutrients. Of the conditionally essential genes, 33.3% and 20.6% were involved in nucleotide metabolism and amino acid metabolism, respectively. Interestingly, *purA*, *purB*, *purC*, *purD*, *purF*, *purH*, *purL*, *purM*, *purN* and *purQ* were found to be essential in RPMI (a synthetic medium for plasma) and SNM3 (a synthetic nasal medium). Indeed, Connolly et. al showed that *purA* and *purB* are essential for growth of JE2 in human and rabbit blood, and pathogenesis in a zebrafish embryo infection model [72]. They further demonstrated that growth of JE2-*purB* was rescued by the addition of adenine and guanine while that of JE2-*purA* was rescued by the addition of adenine. There was a total of eleven conditionally essential genes in SNM3 that were involved in amino acid metabolism (**Fig 3C**). *In silico* growth could be rescued for these mutants with the addition of a combination of L-methionine, L-isoleucine, L-aspartate, and L-asparagine.

We previously found that zinc and molybdate supplementation was needed to support *in silico* growth on CDMG2 and that genes involved in molybdate transport were non-essential in complex medium (likely containing a large range of nutrient types). Here, we identified the conditionally essential genes for growth in CDMG2 and mapped them to their protein structures. Exploiting the cofactor annotation that accompanies experimentally derived protein structures, we found that zinc is a cofactor for ten conditionally essential proteins. Zinc-binding domains were close to the active site or within the substrate binding site for five of the conditionally essential protein structures including *accA* and *accD* [73], *thrS* [74], *pyrC* [75], and *macro* [76]. The activity level of protein structures may be affected by the metal bound at their active site (*e*.*g*., *pyrC* [77]). This evidence tentatively suggests that either zinc is present in trace amounts in CDMG2, or that zinc is not essential for growth.

### Effect of D-glucose on cellular growth and flux distribution

Here, we demonstrate a case of integrating an omics dataset with *i*YS854 to analyze the effect of the addition of D-glucose to the extracellular medium on the intracellular metabolic flux state. Once a model is reconstructed and validated, a condition-specific GEM can be built by constraining the model further using values obtained from experimental measurements. A condition-specific GEM differs from the baseline GEM in that it has a reduced solution space and simulates a flux state that is more representative of the cell’s metabolic state under the tested culture conditions.

#### Condition-specific GEMs agree qualitatively and quantitatively with experimental measurements

**Fig 4:**
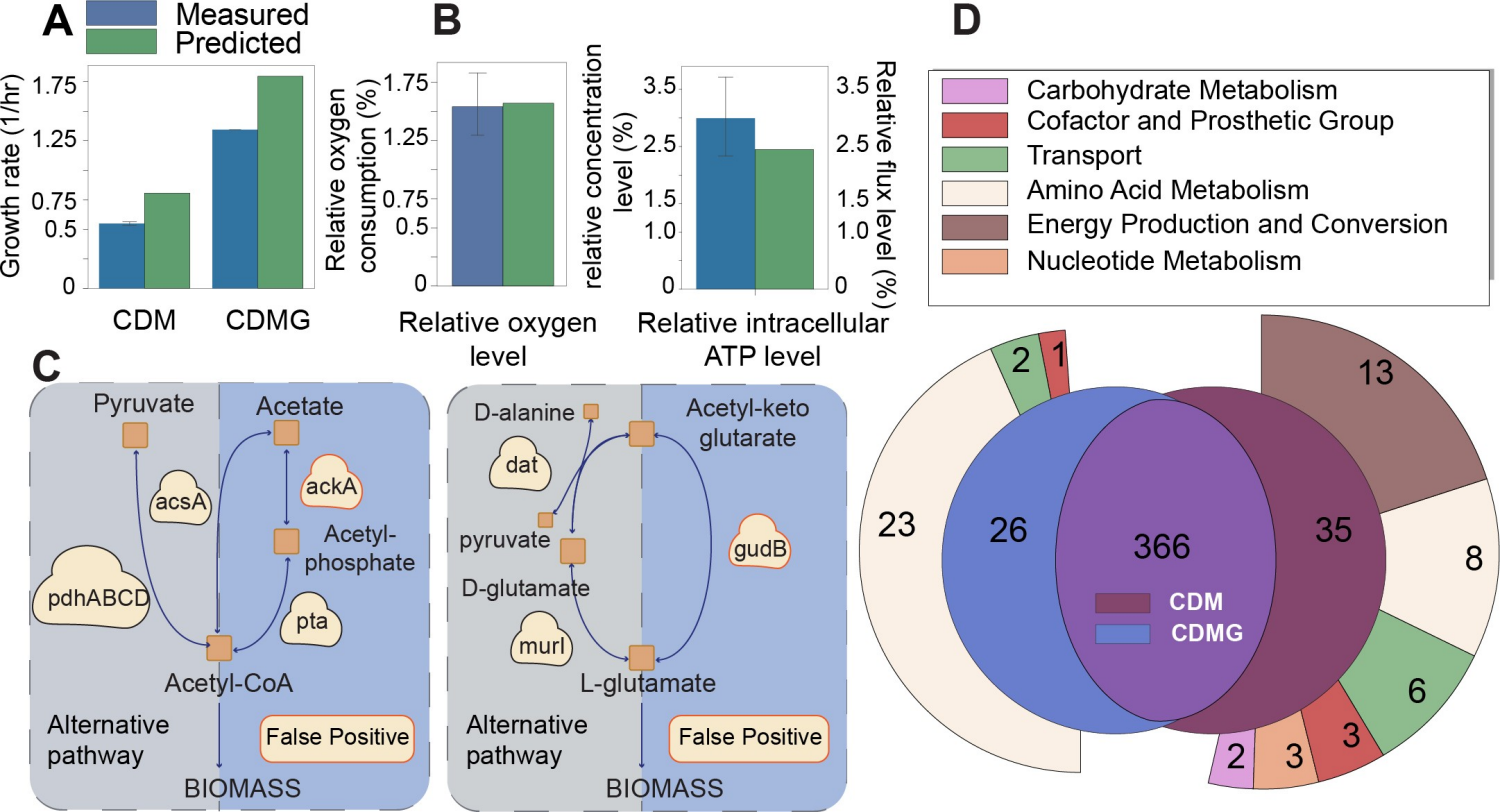
Condition-specific GEM validation and evaluation. (A) Quantitative exo-metabolomics measurements (for both CDM and CDMG) were used to build two condition specific GEMs. Growth was simulated using flux balance analysis and cross-checked against experimentally observed growth rates. See **S1 Appendix** for comparison of predicted and measured relative growth rate across the two media types. (B) We compared the measured and simulated relative oxygen consumption as well as the relative intracellular ATP concentration between the two condition-specific GEMs **(Methods)**. (C) Growth phenotype predictions for 28 CDM-specific mutant GEMs were compared against experimentally observed transposon mutant growth phenotypes. Two genes (*ackA* and *gudB*) were classified as false positives due to the presence of alternative pathways. (D) We simulated single reaction knockouts and compared essential reactions across conditions. The Venn diagram highlights the differences in reaction essentiality between the CDM-specific GEM and the CDMG-specific GEM.

We queried published quantitative exo-metabolomics measurements at four time points for cultures of *S*. *aureus* str. JE2 on CDM and CDMG [39]. Both media contain 18 of the 20 amino acids (excluding L-asparagine and L-glutamine), seven vitamins and trace metals but differ in D-glucose content (which is absent in CDM but present in CDMG). We first calculated uptake and secretion rates in both conditions and used them to set additional constraints to the baseline GEM **(Fig S4 in S1 Appendix, Methods, S10 Table in S2 Appendix)**. The uptake rates for L-proline, glycine, and L-threonine were highest in CDM while those for D-glucose, L-threonine, and L-aspartate were highest in CDMG. Both acetate and ammonium were secreted at higher rates in CDMG than in CDM. We observed that the two condition-specific GEMs simulated a larger maximal growth rate than experimental observation **(Fig 4A)**. Such discrepancies can be attributed to: 1) non-metabolic ATP requirement for cell division, replication and macromolecular polymerization (which are not accounted for in *i*YS854) [15] and; 2) carbon dioxide excretion (for which we have no experimental measurements). To account for these discrepancies, we calculated a growth associated maintenance of 39.92 mmol/gDW/h and non-growth associated maintenance of 3.63 mmol/gDW/h **(Methods, Fig S5 in S1 Appendix).** However, a larger number of data points would yield more accurate values of GAM and NGAM. Next, we calculated the ratio of oxygen consumption and intracellular ATP concentration across the two GEMs and found that the ratios agree with experimental measurements **(Methods, Fig 4B)** [39].

#### Conditional gene essentiality highlights pathways under transcriptional regulation in *S*. *aureus*

We validated gene essentiality predictions against the growth phenotype for 29 transposon mutants cultured on CDM [39]. Of these, seven were essential, eleven were non-essential, and eleven were found to have an intermediate effect on growth. Predictions were made correctly for all of the non-essential genes and all of the essential genes except for *gudB*, which encodes for the oxidative deamination of acetyl-ketoglutarate to glutamate and *ackA*, which encodes for an acetate kinase. L-glutamate biosynthesis can be achieved *via* two metabolic routes; the first involves *gudB*, and the second emcompasses D-alanine transaminase (*dat*—which converts D-alanine to D-glutamate), and glutamate racemase (which catalyzes the isomerization of D-glutamate to L-glutamate). Similarly, acetyl-CoA can be generated *via* several routes including *pta/ackA*, *pdhA/pdhB/pdhC/pdhD*, and *acsA* (**Fig 4C**). The inability of a mutant to grow when it has alternative metabolic routes may be due to reaction kinetic properties. Results for the eleven mutants that exhibited an alternative phenotype revealed cases of isozymes having evolved to function in specific metabolic modules **(S1 Appendix)**.

#### Glucose causes significant changes in the flux solution space

**Fig 5:**
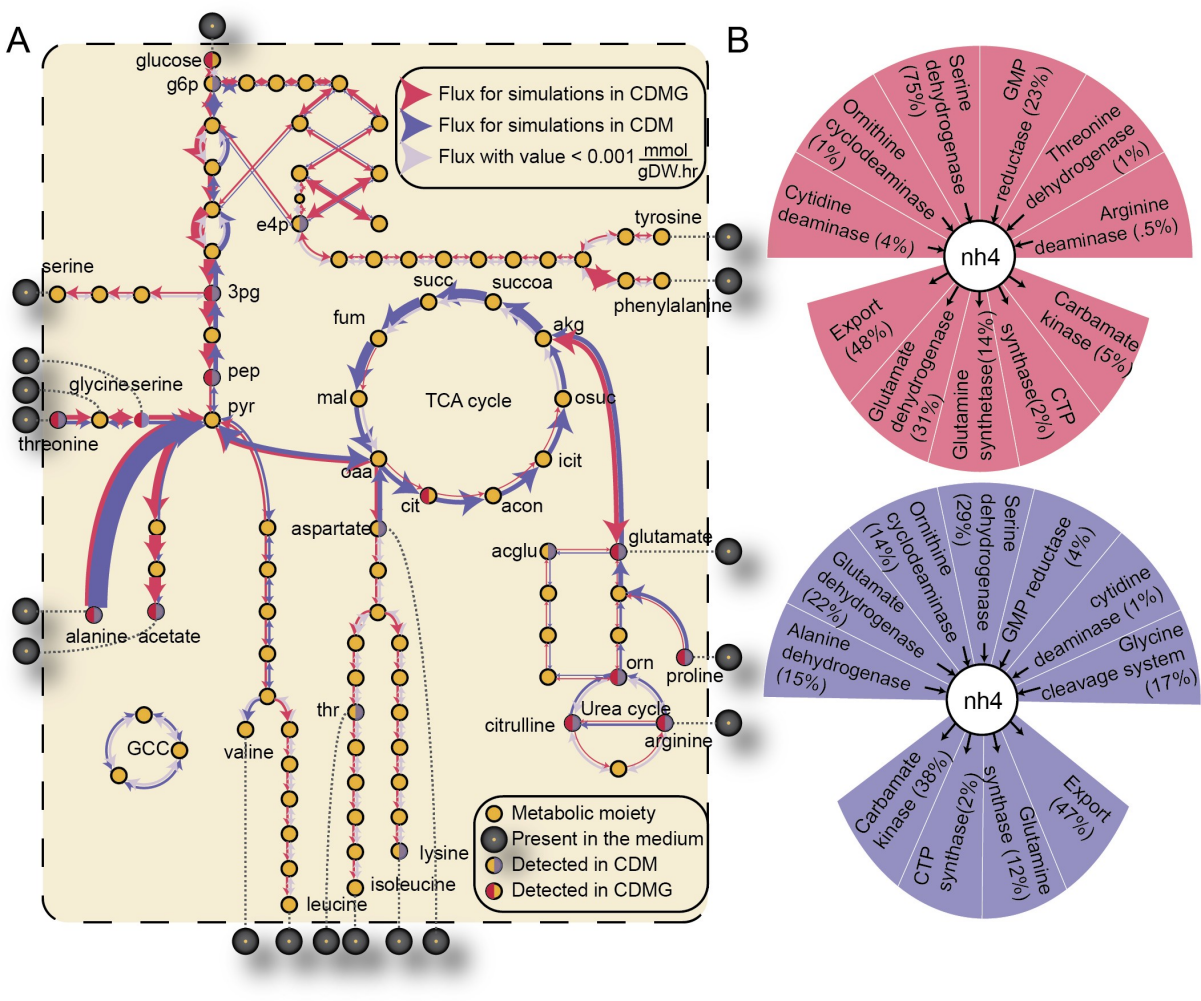
Comparison of flux distribution across two condition-specific GEMs. (A) The reactions that were shown to significantly differ in their flux distribution (determined by the Kolmogorov-Smirnoff test) between the CDMG- and CDM-specific GEM are shown. The width of the arrows qualitatively represents the median flux value across 10,000 sampled fluxes. The blue arrows represent the flux simulation results for the CDM-specific GEM and the red arrows represent the flux simulations of the CDMG-specific GEM. The13C labelled intracellular metabolites detected by NMR in both conditions are highlighted in red (metabolic intermediates derived from extracellular glucose in CDMG) and in blue (metabolic intermediates derived from nine extracellular amino acids in CDM) [79]. Metabolites highlighted in grey are present in the extracellular medium (note that D-glucose is only present in CDMG). (B) Differential cycling of ammonium (as well as several cofactors) between the two GEMs highlights the relative contribution to the production and consumption of ammonium for all reactions utilizing or synthesizing ammonium in CDM (blue) and CDMG (red). Several metabolic processes contribute to the ammonium pool in CDM (including the glycine cleavage system) while serine dehydrogenase is the main source of ammonium in CDMG.

When comparing the two condition-specific GEMs, we found that 148 reactions differed significantly in their flux distribution (p < 0.01, KS test, **Methods**) across both conditions (**Fig 5**). These reactions were predominantly associated with amino acid metabolism (52 out of 148) and energy production and conversion (41 out of 148). Reaction essentiality also varied with 26 and 35 reactions uniquely essential in CDMG and CDM, respectively, while 366 reactions were essential in both conditions (**Fig 4D**). Interestingly, 88% (23 out of 26) of the reactions that were uniquely essential in CDMG were part of amino acid biosynthesis pathways such as branched chain amino acid biosynthesis and aspartate biosynthesis, and were predicted to carry larger median fluxes (**S2 Appendix**). In a transcriptomic analysis of JE2 strains cultured in CDM+galactose, the authors similarly noted a significant upregulation of genes involved in branched amino acid biosynthetic genes. This observation supports our predictions since genes tend to have a higher mean mRNA expression level when they are essential [78]. Conversely, 37% (13 out of 35) of the reactions that were only essential in CDM but not in CDMG were involved in energy production and conversion. Thus, as a result of constraining the uptake and secretion rates alone, flux balance analysis indicates that upon addition of glucose to the medium, isolates utilized amino acids from the medium in conjunction with synthesizing amino acids *de novo*. However, when glucose was subtracted from the medium, a lower growth rate was observed, and the cells were predicted to utilize the available amino acids in the medium towards energy production (*via* gluconeogenesis) and protein biosynthesis (for biomass production). Additionally, the cells relied on nine more metabolic processes to achieve growth **(Fig 4D)**.

The CDM-specific model carried larger median fluxes across reactions of the TCA cycle and gluconeogenesis than the CDMG-specific GEM. Indeed, the published NMR peak intensity spectrum of JE2 cultured in CDM supplemented with 13C-labeled amino acids indicated that gluconeogenic intermediates including D-glucose-6-phosphate, D-glucose-1-phosphate, 3-phospho-D-glycerate, and acetate are produced from a variety of extracellular amino acids including L-glutamate, L-proline, and L-arginine. Thus, in CDM cultures, gluconeogenic intermediates are produced from various extracellular amino acids (**Fig 5A**). In contrast, the CDMG-specific GEM carried larger median fluxes in amino acid biosynthetic pathways, the PPP, and upper and lower glycolysis. Again, the NMR peak intensity spectrum for CDM supplemented with 13C-labeled D-glucose revealed that several amino acids including L-alanine, L-glutamate, L-arginine, L-aspartate, L-asparagine, L-proline, and L-glutamine as well as acetate (a glycolytic end product) were being synthesized from extracellular glucose [39].

#### Condition-specific GEMs reveal that the sole addition of glucose causes a reorganization of cofactor cycling

We next analyzed the predicted cycling of ammonium and major cofactors including NADP and ATP. For each metabolite, we compared the relative contributions of all producing and consuming reactions between the two conditions. We found that ammonium cycling was drastically different across both models with the main source of ammonium being serine dehydrogenase (carrying 75% of the biosynthetic flux producing ammonium) in CDMG while multiple reactions contributed to the pool of ammonium in CDM. The glycine cleavage system produced 17% of the ammonium in CDM and carried a significantly higher median flux (1.063 mmol/gDW/hr as opposed to 0.436 mmol/gDW/hr; **Fig 5B)**.

Similarly, we found that overflow pathways contributed a larger percentage to the pool of ATP in CDMG (with 54%, 29%, and 16% being produced by ATP synthase, phosphoglycerate kinase, and acetate kinase, respectively) than in CDM (82% and 18% of the ATP was produced by ATP synthase and succinyl-coa synthase, respectively). These differences are a direct result of the larger acetate secretion constraint in CDMG which forced the metabolic flux to be redirected away from the TCA cycle to acetate synthesis. The NADPH pool, which is partially recycled (28%) by the TCA cycle (as by-product of decarboxylating isocitrate to oxalosuccinate) in CDM, is predominantly recycled (94%) *via* the PPP in CDMG. NADPH is an important cofactor for the biosynthesis of fatty acids, nucleic acids, and amino acids and the CDMG-specific GEM predicted a larger total flux through NADPH. This prediction is consistent with published experimental measurements of the NADP+/NADPH ratio [55]. Taken together, these findings suggest that in the presence of high concentrations of glucose, *S*. *aureus* relies more heavily on glycolysis for its energy generation and utilizes D-glucose as a main source for the synthesis of a large range of biomass precursors.

## Discussion

This study presents the most recent and up-to-date genome-scale metabolic reconstruction for the gram-positive pathogen *S*. *aureus*. We validated *i*YS854 both quantitatively and qualitatively against a variety of data sets and observed a significant improvement with respect to the starting model in both carbon catabolic capabilities as well as gene essentiality prediction. We then integrated time course quantitative exo metabolomics with *i*YS854 to analyze the effects of exogenous glucose on the intracellular flux distribution.

Inconsistencies of model-driven predictions with *in vitro* observations highlighted gaps in knowledge as well as non-essential biomass components (including cell wall components, haem, and menaquinone). Interestingly, cell wall deficient strains are involved in persistence *in vivo* [80], while *menD* mutants exhibit the small colony variant (SCV) phenotype, a phenotype known to be associated with increased persistence and resistance to antibiotics *in vivo*. Taken together, these results hint towards an altered biomass composition as a result of exposure to environmental stresses such as antimicrobials.

Gene essentiality predictions on synthetic physiological media and chemically defined media revealed the essentiality of purine, pyrimidine, and amino acid biosynthesis for growth under nutrient limited conditions. Nucleotide biosynthesis, which was predicted to be essential in RPMI and SNM3, has been shown to be essential for growth in blood for a variety of bacteria including *S*. *aureus*, *E*. *coli*, *Salmonella*, and *B*. *anthracis* [72,81]. Additionally, iYS854 predicts amino acid biosynthesis to be essential in SNM3 (a synthetic nasal medium), and our results were confirmed experimentally by Krismer et. al [53].Together, these findings point towards putative antibiotic targets for the treatment of bloodstream and nasal infections.

Elevated concentrations of blood glucose is common across diabetic patients and *S*. *aureus* is the most frequently isolated and virulent pathogen from diabetic foot infections [82]. Condition-specific models revealed drastic systems-level differences in flux distributions across strains when they were exposed to D-glucose. For example, despite the availability of amino acids in the medium, *S*. *aureus* was predicted to utilize both extracellular glucose and amino acids towards protein production in order to satisfy its biomass requirements. This finding is supported by the observation that genes involved in amino acid biosynthesis are highly expressed when D-glucose is added to the medium [55] and hints towards a kinetic constraint favoring the uptake and utilization of extracellular glucose over that of extracellular amino acids towards amino acid biosynthesis. Indeed, *S*. *aureus* strains express four glucose transporters suggesting that together, they can induce high levels of glycolytic flux [83].

The addition of glucose to the medium induced significant metabolic rewiring, with production of ATP switching from the Krebs cycle to overflow pathways as evidenced by the large acetate secretion rate. Significantly higher glycolytic fluxes were predicted in the presence of glucose. Aerobic fermentation also occurs in *E*. *coli* when the glucose consumption rate is large, and the cell cannot reoxidize reduced equivalents at a sufficient rate [84]. Importantly, glycolytic activity exhibited by *S*. *aureus* strains has been shown to induce hypoxia inducible factor 1α signalling and promote the proinflammatory response to infection [85]. The absolute consumption of oxygen was predicted and experimentally shown to be higher in the presence of glucose (as confirmed by experimental evidence [39]). We also predicted an elevated flux through the PPP in the presence of glucose, which was confirmed by an experimentally observed higher NADP/NADPH ratio [55]. In agreement with our predictions, the inactivation of the TCA cycle was found to cause an increase in the carbon flow across the PPP in *S*. *aureus* [86]. Here we show that the generation of NADPH is mediated by both the PPP and the TCA cycle and that the increased flux through the PPP compensates for the decreased flux in the TCA cycle.

Our results demonstrate that the updated *S*. *aureus* GEM, *i*YS854, accurately captures experimentally measured differences in central metabolism in the presence and absence of glucose and that the importance of metabolic modules changes drastically under different *in silico* physiological growth media. This study is a first step towards understanding the systems-level metabolic response of *S*. *aureus* to differing media compositions from a constraint-based modeling perspective.

## Materials and methods

### Modifications to the metabolic network

A draft core *S*. *aureus* GEM was built by taking the common reactions between the *E*. *coli* core GEM [87] and the starting *S*. *aureus* GEM [11] and adopting the *E*.*coli* core biomass objective function (BIOMASS_Ecoli_core_w_GAM) [88]. We curated the network after reviewing literature using the COBRApy toolbox (**Tables S1, S2 and S3 in S2 Appendix**) [89]. Modifications included reaction, gene and metabolites addition/removal, and annotations of reactions and genes with confidence scores, references and metadata. We assigned confidence scores as per the standards set by Thiele et. al [90] and novel instance IDs as per BiGG standards [91]. We downloaded the genomic sequence for *S*. *aureus* str. JE2 from NCBI (accession number CP020619.1). Genes were updated with names as assigned in the literature (when available) or as generated during automatic genome annotation. We added the E.C. numbers obtained from the genome annotation as metadata to the modelled genes. We then downloaded the *S*. *aureus* Swiss-prot knowledge base (which contains manually reviewed proteins and protein metadata specific to *S*. *aureus*) and cross-referenced the modelled genes with Swiss-prot IDs using bi-directional best BLAST (PID > 80%, e-value< 10^−3^) [92,93].

### Flux simulations and network evaluation

One of the crucial steps involved in a reconstruction is the evaluation of the network flux carrying capability. In addition to ensuring the successful production of biomass precursors, we examined some general properties of the flux distribution. The starting model could not simulate flux through the full TCA cycle and erroneously simulated the dissipation of 13 energy carriers when all exchanges were closed including ATP, CTP, GTP, UTP, ITP, NADH, NADPH, FADH2, FMNH2, MQH7, acetyl-coa, L-glutamate and intracellular proton. Such an aberration is commonly found to be caused by a set of reactions constituting together erroneous stoichiometrically balanced energy generating cycles (ECGs) [23]. To search for energy generating cycles we followed the workflow established by Fritzemeier et. al [94]. Briefly, we blocked all extracellular exchanges by constraining the upper and lower bounds to 0 and iteratively added 14 energy dissipation reactions **(S4 Table in S2 Appendix)**. An energy dissipation reaction is a reaction that consumes high energy metabolites. We simulated maximal flux through one dissipation reaction at a time using flux balance analysis (FBA) [95]. Energy generating cycles existed when the maximal flux through any energy dissipation reaction was larger than 0. We found that EGCs were caused by: 1) sets of reactions carrying out the same function but with inverted reversibility, 2) the inclusion of reactions that are not known to occur in *S*. *aureus* nor have any genetic basis for their inclusion (such as 2-oxoglutarate synthase and fumarate reductase allowing reductive TCA), and 3) reversible reactions that could generate energy carrying moieties when the flux was running in the reverse direction. As a result of removing and adjusting the network accordingly, iYS103 successfully simulated flux through the TCA cycle and could not freely charge any of the 13 high energy carriers (**Fig S1 in S1 Appendix**). The final core network contains 103 unique ORF assignments, 70 metabolic processes and 58 metabolic species and can successfully simulate the utilization of the Krebs cycle.

### Addition of gene and reaction metadata

Reactions were annotated with COG subsystems following the same classification scheme as previous GEM reconstructions [70,91]. The subsystems consisted of: 1) amino acid metabolism, 2) carbohydrate metabolism, 3) cell wall and membrane metabolism, 4) cofactor and prosthetic group metabolism, 5) energy production and conversion, 6) transport, 7) nucleotide metabolism, 8) lipid metabolism and 9) inorganic ion transport and metabolism. We also added as a note to each metabolic reaction the metabolic sub-module that it is described to participate in throughout the literature. We annotated metabolic reactions with 65 metabolic sub-modules. To visualize the amount of novel content added to each metabolic subsystem, we compared the updated metabolic gene content with the metabolic gene content across the 4 previous metabolic reconstructions. Genes were then classified in sub-modules according to the metabolic reactions they participate in. For each sub-module, a fraction representing the ratio of novel genes to the total number of genes it contains was computed. A gene was considered “novel” when it was not accounted for in the previous reconstruction **(Fig 2A)**.

### Addition of structures and structure-guided reconstruction

The structural systems biology (ssbio) pipeline was run to map crystallized 3-dimensional structures of proteins deposited in the Protein Data Bank (PDB) to the genes included in the genome scale reconstruction [48,49]. A blast cutoff was chosen at 70%. Genes that could not be mapped through this method to a crystal structure were mapped to their nearest homolog with an existing structure **(S4 Table in S2 Appendix)**. Homology models were built from this template and subsequently modified to match the amino acid sequence of the USA300 query protein **(S5 Table in S2 Appendix)**.

### Biomass objective function

We adapted the weight fractions for the 5 polymer categories and the pool of solutes from Heinemann et. al [12]. The authors computed a biomass composition by averaging experimentally derived weight fractions across several *S*. *aureus* strains grown in different media conditions. We proceeded to compute the relative ratios of the DNA precursors using the *S*. *aureus* genomic sequence and the RNA and protein weight fractions using transcriptomics data derived for *S*. *aureus* str. plasmid cured LAC (JE2) grown on a chemically defined medium with galactose as the main gluconeogenic nutrient source [96]. Computations were performed *via* BOFdat, a python package for biomass objective function derivation [51]. We included amino acids in their tRNA bound form because two of the twenty amino acids are only synthesized while complexed with tRNA [97]. The relative quantities for the cell wall precursors and lipids were adapted again from Heinemann et. al. However, the updated metabolic network includes the biosynthesis of downstream precursors for some of the cell wall precursors. For example, we replaced the peptidoglycan monomer with a wall teichoic acid bound peptidoglycan dimer, and lipoteichoic teichoic acids with charged lipoteichoic acids. We adjusted the relative coefficients according to the replaced precursor’s molecular weight. The pool of solutes was adapted from [52] and updated with metals and trace molecules (chosen based on literature evidence [98] and protein cofactor utilization obtained from the metadata associated with the 3-D protein structures; **S7 Table in S2 Appendix)**.

### Growth carrying capability of multiple media types and prediction of necessary supplementations

We modelled growth on a defined medium by setting the lower bound to the reactions exchanging metabolites that are present in the medium to -10 mmol/gDW/hr. A negative value signifies exchange from the medium to the cell. The lower bound to all other exchanges was set to 0 mmol/gDW/hr. The simulated media types are available in **S8 Table in S2 Appendix.** When growth could not be achieved, we searched for minimal medium supplementations needed to support growth. For that purpose, we changed the objective of the optimization problem to the minimization of the number of additional open exchange reactions and constrained flux through the biomass objective function to a minimal value of 1 *hr*^−1^ **(S9 Table in S2 Appendix)**. We set the lower bound to all exchange reactions to -10, and the solver configuration tolerance feasibility to 10^−9^ using COBRApy.

$$min\sum_{j}y_{j}\;\forall_{j}\in Subset\;of\;exchange\;reactions$$ (1)

$$\sum_{\forall_{i}\in Metabolites}S_{ij}.v_{j}=0\;\forall_{j}\in Reactions$$ (2)

$$y_{j}.LB_{j}\leq v_{j},\;\forall_{j}\in Subset\;of\;exchange\;reactions$$ (3)

$$v_{biomass}=1\;{hr}^{-1},$$ (4)

$$y_{j}\in (0,1)$$

Aerobic environments were simulated by setting the lower bound for oxygen exchange to -20 mmol/gDW/hr. Oxygen exchange was blocked to simulate an anaerobic growth environment. The utilization of nitrate as an alternative electron acceptor was simulated by setting the lower bound for nitrate exchange to -20 and the lower bound for oxygen exchange to 0.

### High throughput BIOLOG phenotypic array

Model benchmarking on carbon sources was performed using Biolog plates PM1 and PM2 (BIOLOG Inc. Hayward, CA). The recommended protocol was followed as described by (Zuniga et al., 2016), with the following modifications. *S*. *aureus* USA300 was grown to mid-log phase in modified TSB media, pelleted *via* centrifugation at 4,000 x g for five minutes, washed and resuspended in fresh media to a final OD = 0.1. Aliquots of 100 uL were inoculated into Biolog plates and examined in the plate reader at time zero, then each hour from 1–12 h, and finally at 24 h. Plates were housed in a plate reader under sterile conditions. The plates for both the PM1 and PM2 plate (carbon sources) were run at 490 nm to examine dye absorbance alterations and 750 nm to assess optical density. M9 minimal medium supplemented with niacin and thiamin was used as the minimal medium to simulate for the utilization capability of 68 carbon sources. The simulation results were then compared against experimental observations **(S10 Table in S2 Appendix)**.

### Gene essentiality prediction

The predicted mutant growth phenotypes were obtained by simulating a gene knockout using the cobra.flux_analysis.single_gene_deletion command. The mutants were cultured on tryptic soy broth (TSB, a rich and complex medium for which the composition is unknown) and the observed gene essentiality for this condition was reported. To mimic TSB, we simulated growth by allowing inward flux of all the extracellular nutrients included in the reconstruction. We set the objective function to BIOMASS_iYS_reduced (which excludes the pool of measured intracellular solutes detected by NMR for growth of *S*. *aureus* on CDM+glucose). A gene was deemed to be essential when its knockout resulted in a maximal growth of less than 0.0001 hr^-1 or when the solution status was not optimal (**S11 Table in S2 Appendix**).

To interrogate the capability of iYS854 to recapitulate the mannitol fermentation capability across mutants, we first confirmed that the model could simulate growth on mannitol in an oxygen depleted environment by allowing uptake of mannitol, M9 minimal medium components, thiamin and niacin. Extracellular oxygen exchange was blocked to mimic the anaerobic environment. We subsequently assessed gene essentiality by using the cobra.flux_analysis.single_gene_deletion command and compared the results against experimental observation. In order to assess the GEM’s capability to predict pigment formation, we set the production of staphyloxanthin as the objective of the maximization problem. Growth on rich medium was then simulated by allowing inward flux across all exchanges. Again, we determined gene essentiality to assess the effect of gene knockouts on the production of staphyloxanthin (**S12 Table in S2 Appendix**). Gene essentiality on all other media types was determined by setting the lower bound to exchange reactions to -10 when they imported a metabolite that was present in the medium (**S13 Table in S2 Appendix**).

### Cell weight measurements

Single colonies of *S*. *aureus* str. LAC were inoculated into 5 mL Roswell Memorial Park Institute (RPMI) 1640 supplemented with 10%LB (RPMI+10%LB) and incubated overnight at 37oC with rolling. Overnight cultures were diluted into tubes containing 18 mL fresh media to a starting OD_600_ 0.01 and incubated at 37oC with stirring until cultures OD_600_ 0.4. Precultures were diluted back into 6 new tubes, containing 20 mL fresh media to OD_600_ 0.01 and growth was monitored until cultures reached OD_600_ 0.5. The 6 tubes were mixed in baffled flask and OD_600_ was taken. Preweighed 0.2 μm filter was placed into a clean glass filter holder above. 40 mL culture was passed through filter and unit was washed with 15 mL ddH_2_O. A final OD_600_ reading was taken from remaining culture. Filter disc was transferred to a clean petri dish placed in incubator at 80oC ON. The next day, filter discs were acclimated to room temperature for 45 min and reweighed. Dry cell weights were taken as the average of three weight measurements. We obtained an average dry cell weight of 9.6 mg at an OD600 of 0.58 (**S14 Table in S2 Appendix**).

### Condition-specific GEMs were built using time course quantitative exo metabolomics

Hasley et. al reported absolute concentration measurements for extracellular ammonium, acetate, glucose and all 18 amino acids and complemented these measurements with corresponding time-course OD readings. We calculated the growth rate and uptake rates in both conditions as specified below:

SUR=μ×m, (5)

$$\mu =slope({log}_{gDW},t)\;for\;t\in (0,2,4,6,8)$$ (6)

m=slope([X],gDW) (7)

Where [X] is the set of concentration measurements across t in mmol/L, gdW is the gram dry weight in g/L, t is the time in hours, *μ* is the growth rate, SUR is the substrate uptake rate in *mmol*/*t*/*gDW*.

For each condition, we verified that the uptake and secretion rates were mass balanced and that the overall flux of elements going towards biomass production (i.e. the total influx minus the total outflux) is larger than the total flux of elements needed to support biomass production at the experimentally measured growth rate.

$$\sum_{e,i}N_{e,i}*{SUR}_{i}-\sum_{e,j}N_{e,j}*{SUR}_{j}>\sum_{e,k}P_{e,k}*b_{k}*\mu >0$$ (8)

∀e∈{C,H,P,O,N,S},

∀i∈exchangesallowingnutrientsinflux,

∀j∈exchangesallowingnutrientsoutflux

∀k∈biomassprecursors

Where *N*_*e*,*i*_ is the base ratio for element *e* in the metabolic structure for nutrient *i*, *SUR*_*i*_ is the substrate uptake rate for nutrient *i*, *b*_*k*_ is the relative coefficient for the biomass precursor *k*, *P*_*e*,*k*_ is the base ratio of element *e* in the metabolic structure of the biomass precursor *k*.

We proceeded to build two condition-specific GEMs by constraining the reactions exchanging the extracellular nutrients to +/- 10% of the measured corresponding calculated uptake and secretion rates (**S15-S17 Tables in S2 Appendix**). We subsequently ran flux balance analysis (FBA) to simulate maximal biomass production.

### Growth associated maintenance calculation

We calculated a theoretical growth associated maintenance by changing the objective for both condition-specific GEMs to ATP production (*i*.*e*., the objective coefficient for the ATP maintenance reaction was set to 1). The maximal flux through the ATP maintenance reaction obtained was 1.93 *mmol*/*gDW*/*h* for the CDM-constrained GEM and was 12.64 *mmol*/*gDW* for the CDMG-constrained GEM. As a result, we obtained a growth associated maintenance of 9.59 *mmol*/*gDW*/*h* (which is the slope of the line obtained from plotting maximal flux through ATPM against growth rate) and a non-growth associated maintenance of −5.87 *mmol*/*gDW*. Since non-growth associated maintenance should be positive, we hypothesize that data-sets {*v*_*ATPM*_,*μ*} covering a larger range of conditions (e.g. anaerobic conditions as well as alternate carbon/nitrogen sources) coupled with measurements of CO2 secretion rates is needed to lead to more accurate values. We instead used the GAM and NGAM values experimentally obtained for *E*. *coli* and iteratively computed maximal growth for decreasing percentages of the initial value. For each condition, the GAM and NGAM value is chosen when the simulated maximal growth corresponds to the observed experimental growth rate **(S1 Appendix)**.

### Calculation of a proxy for relative intracellular ATP concentration and relative oxygen level

We sampled the steady state flux space a total of 10,000 times using the cobra.flux_analysis.sample() command from the cobrapy package. To obtain a proxy for the predicted relative intracellular ATP concentration we calculated the ratio of the sum of all metabolic fluxes producing ATP across both condition:

$$rATP_{\frac{CDM}{CDM+glucose}}=\frac{median({\sum_{i=1}^{n}\;a_{ATP}}_{}*v_{i}{)}_{s1}}{median(\sum_{j=1}^{m}\;a_{ATP}*v_{j}{)}_{s2}}\;\forall i\in R_{1},\forall j\in R_{2},\forall s_{1}\in S_{1},\forall s_{2}\in S_{2}$$ (9)

where *R*_1_ are the set of reactions yielding ATP and *S*_1_ is the set of 10,000 sampled fluxes in the CDM-specific GEM, *R*_2_ is the set of reactions yielding ATP and *S*_2_ is the set of 10,000 sampled fluxes in the CDMG-specific GEM, *a*_*ATP*_ is the stoichiometric coefficient for ATP in reaction *i*, and *v*_*i*_ is the calculated flux in *mmol*/*gDW*/*hr* through reaction *i*, and the median is taken across 10,000 samples.

Similarly, we computed a proxy for the relative oxygen level by computing the relative flux for the oxygen exchange:

$$rO2_{\frac{CDM}{CDM+glucose}}=\frac{median(v_{O2}{)}_{s1}}{median(v_{O2}{)}_{s2}}\;\forall s_{1}\in S_{1},\forall s_{2}\in S_{2}$$ (10)

Where *v*_*O*2,*i*_ is the flux through EX_o2_e in *mmol*/*gDW*/*hr* and the median is taken across 10,000 sampled fluxes.

### Assessment of significant differences in flux distribution

Flux balance analysis was run in both conditions and the fluxes were sampled 10,000 times. All reaction fluxes were normalized by dividing by the growth rate to account for growth differences across the two media types. The flux distribution for each metabolic process was compared across both conditions using the Kolmogorov-Smirnov test, a non-parametric test which compares two continuous probability distributions. For this purpose we used the command scipy.stats.ks_2samp from the scipy package. The distribution across two reactions was deemed to be significantly different when the Kolmogorov-Smirnoff statistic was larger than 0.99 with an adjusted p-value < 0.01. We proceeded to plot the reactions highlighted in this process in Fig 5.

## Supporting information

S1 Appendix 1. Modification of the network content. 2. MRSA-specific additions reveal metabolic knowledge gaps. 3. False negatives highlight gaps of knowledge in metabolism. 4. Gene essentiality highlights cases of non-essential protein complex subunits. 5. Protein structures coupled with gene essentiality uncover false and real isozymes. 6. Mutants with partially affected growth corroborate the presence of functional isozymes in *S*. *aureus*. **Figure S1:** Comparison of gene essentiality prediction accuracy across different *S*. *aureus* GEMs. **Figure S2:** Cell wall biosynthesis false negatives. **Figure S3:** Gene essentiality predictions for growth of *S*. *aureus* strains on multiple media types. **Figure S4:** Uptake rates for the top 10 extracellular metabolites calculated from the absolute quantitative exo-metabolomics measurements for growth of *S*. *aureus* strain LAC on chemically defined medium (CDM) and glucose + chemically defined medium (CDMG).
(PDF)

S2 Appendix **Table S1**: iYS854 metabolic reactions. **Table S2:** iYS854 metabolic genes. **Table S3**: iYS854 metabolites. **Table S4**: Energy dissipation reactions tested. **Table S5**: iYS854 protein structures, mapped to PDB crystal structures using the ssbio package. **Table S6**: iYS854 protein structure homology models generated using the ssbio package. **Table S7**: Biomass objective function. **Table S8**: Simulated chemically defined media type. **Table S9**: Growth predictions and supplementation on defined minimal media. **Table S10**: High throughput microarray growth phenotypes for aerobic conditions aligned with experimental predictions. **Table S11**: Prediction and validation of transposon mutant phenotypes. **Table S12**: Gene essentiality prediction for 854 gene knockout mutants simulated on rich medium. **Table S13**: Gene essentiality prediction for 854 gene knockout mutants on chemically defined minimal media. **Table S14**: S. aureus dry cell weight measurements. **Table S15**: Uptake rates calculated from the absolute quantitative exo-metabolomics measurements for growth of S. aureus strain LAC on chemically defined medium (CDM) and glucose + chemically defined medium (CDMG). **Table S16**: Elemental mass balance for the two sets of exo-metabolomics. **Table S17**: Upper and lower bounds in the condition-specific genome scale models. We allowed for a variation in +/- 15% of the computed uptake rate.
(7Z) Click here for additional data file.

S1 Data iYS854 –*S. aureus* genome-scale metabolic reconstruction (complete). (JSON)

S2 Data iYS103 –*S. aureus* genome-scale reconstruction of core metabolism (includes glycolysis/gluconeogenesis, TCA cycle, respiratory pathway, glutamate metabolism, pentose phosphate pathway, transport and core biomass reaction). (JSON)
