# Supplementary material for: A computational knowledge-base elucidates the response of Staphylococcus aureus to different media types
Source: PLoS Comput Biol. 2019 Jan 9;15(1):e1006644. doi: 10.1371/journal.pcbi.1006644 (PMC6326480; doi:10.1371/journal.pcbi.1006644)
Supplement: S1 Appendix — 1. Modification of the network content. 2. MRSA-specific additions reveal metabolic knowledge gaps. 3. False negatives highlight gaps of knowledge in metabolism. 4. Gene essentiality highlights cases of non-essential protein complex subunits. 5. Protein structures coupled with gene essentiality uncover false and real isozymes. 6. Mutants with partially affected growth corroborate the presence of functional isozymes in S. aureus. Figure S1: Comparison of gene essentiality prediction accuracy across different S. aureus GEMs. Figure S2: Cell wall biosynthesis false negatives. Figure S3: Gene essentiality predictions for growth of S. aureus strains on multiple media types. Figure S4: Uptake rates for the top 10 extracellular metabolites calculated from the absolute quantitative exo-metabolomics measurements for growth of S. aureus strain LAC on chemically defined medium (CDM) and glucose + chemically defined medium (CDMG). (PDF) [file pcbi.1006644.s001.pdf]

Text:

1. Modification of the network content:
2. MRSA-specific additions reveal metabolic knowledge gaps:
3. False negatives highlight gaps of knowledge in metabolism:
4. Gene essentiality highlights cases of non-essential protein complex subunits.
5. Protein structures coupled with gene essentiality uncover false and real isozymes:
6. Mutants with partially affected growth corroborate the presence of functional isozymes in *S. aureus*:

Figures:

S1 Fig: Comparison of gene essentiality prediction accuracy across different *S. aureus* GEMs.
S2 Fig: Cell wall biosynthesis false negatives.
S3 Fig: Gene essentiality predictions for growth of *S. aureus* strains on multiple media types.
S4 Fig: Uptake rates for the top 10 extracellular metabolites calculated from the absolute quantitative exo-metabolomics measurements for growth of *S. aureus* strain LAC on chemically defined medium (CDM) and glucose + chemically defined medium (CDMG)
S5 Fig: GAM and NGAM calculation for *S. aureus*
S6 Fig: Comparison of predicted and measured relative growth rate across the CDM versus CDM+glucose

Text:

1. Modification of the network content:

We started the reconstruction process by removing erroneous content from the starting model. The content removed included gap filling reactions, redundant biochemical networks, erroneous pathways, non-functional isozymes and genes with unknown functions erroneously associated with metabolic processes. For example, *S. aureus* is known to be incapable of *B* oxidation, a pathway that was fully functional in the starting model. These modifications included directionality changes, changes in metabolite identifiers and enzyme cofactor usage. For example, *S. aureus* is known to use NADPH and not NADH for fatty acid biosynthesis (1,2), menaquinol-7 and not menaquinol-8 for respiration and its genome only carries the genes for anaerobic and not aerobic biosynthesis of protoheme. Reactions were set to be irreversible when sufficient supporting literature evidence was available. For example, a recent kinetic and structural analysis of *S. aureus* alanine racemase suggested that the L-alanine to D-alanine direction is favored (3). An irreversible reaction is modelled by setting the lower bound to its flux to 0. Reversibility was assumed when insufficient evidence suggested the opposite and when

the reverse direction did not allow production of an energy currency or an oxidative potential (e.g., ATP, NAD, pumping of a proton to the extracellular matrix, oxygen). We used literature-based evidence to modify gene protein reaction rules (GPR). For example, iron transport is known to be mediated by a number of multi-protein complexes in *S. aureus* (4) and we updated the corresponding GPR to reflect both the redundancy and complexity of the metabolic process.

2. MRSA-specific additions reveal metabolic knowledge gaps:

Accessory metabolic capabilities are capabilities that are not shared by all strains of a species and can confer divergent growth and antibiotic resistant phenotypes (5,6). We thus incorporated known metabolic features specific to the USA-300 group of MRSA strains. For example, the lower sensitivity to spermine toxicity in CA-MRSA strains (7) has been associated with one gene (*speG*) within the arginine catabolic mobile element encoding a spermine/spermidine acetyltransferase. While the full catabolic pathway was added, we could not assign a gene protein reaction rule to each metabolic step due to lack of genomic and literature evidence. We conclude that future work could help to fill the knowledge gaps in the spermidine catabolic pathway. Another gene - *mecA*, which is predominantly present in MRSA strains is known to confer multidrug resistance (8). It encodes a penicillin-binding protein (pbp2A), which constitutes an acquired transpeptidase that functions in concert with two other native transpeptidases (pbp1 and pbp2) (9,10) involved in one of the final steps of the peptidoglycan biosynthetic pathway. As such, the gene-protein-reaction rule (GPR) which maps genes to a reaction was updated to reflect the capability of pbp2A to compensate for pbp2 (which under antibiotic stress is non-functional).

3. False negatives highlight gaps of knowledge in metabolism:

A total of 4 genes involved in the biosynthesis of L-methionine from S-Adenosyl-L-homocysteine (AHCYS) initially fell under the category of false positives. S-Adenosyl-L-homocysteine is a by-product of menaquinone-7 biosynthesis and L-methionine biosynthesis is the only metabolic route known to consume AHCYS. Since the genes involved in L-methionine biosynthesis are not essential, we suggest that there is another unknown pathway for the utilization and conversion of AHCYS in *S. aureus*. To compensate for this known knowledge gap, we added a demand reaction which is an unbalanced intracellular reaction that allows for the consumption of a metabolite (11). Before this addition, the total number of false negatives was 28.

Two genes involved in molybdate uptake (*modA* and *modC*) were observed to be non-essential for growth in LB. It has been shown that mutants defective in *modABC* (the complex involved in molybdate transport) had fully restored molybdate activity under non physiologically high concentrations of molybdate suggesting that there is another non-specific molybdate transport mechanism (12).

4. Gene essentiality highlights cases of non-essential protein complex subunits.

We found 27 cases of non-essential subunits of multiprotein complexes, a phenomenon previously observed in cyanobacteria (13). For example, proton-sodium antiport is mediated by three multi-protein membrane complexes (*mpsABC, mnhABCDEFG (14)* and *mrpABCDEFG (15)*) and at least one gene in each complex is essential and one gene is non-essential for growth in rich medium. All three complexes are involved in respiration but their exact mechanism remains unknown. Gene essentiality observations suggest that their functions are not compensatory. We noticed a similar scenario for other prominent multiprotein complexes: 1) the ATP synthase *atpABCDEHG* (for which only *atpC* and *atpE* were observed to be essential); 2) dihydroxyacetone phosphotransferase *dhaKML* (for which only *dhaL* was observed to be essential); 3) anthranilate synthase *trpGE* (for which only *trpG* was observed to be essential), and: 4) molybdate-binding complex *modAB* (*modA* was observed to be essential). Intriguingly, while the model predicted that *modA* and *cysA* were essential for growth, experimental observations indicated otherwise. Both genes along with *modB* participate in molybdate transport. Molybdate is utilized for molybdenum cofactor biosynthesis and has been shown to be indispensable for the proper function of dependent protein complexes. We hypothesize that either *S. aureus* encodes for other complexes capable of carrying out molybdate transport, or molybdate is not truly essential for growth. In the latter case, we argue that molybdate would again constitute a conditional biomass precursor.

5. Protein structures coupled with gene essentiality uncover false and real isozymes:

In some cases, the genome annotation indicated the presence of homologous genes, many of which were modelled as isozymes. However, the contextualization of gene essentiality with genome scale modelling highlighted cases in which the homologous genes were not isozymes and cases in which one of the two homologous genes encoded for an additional function. In other words, they could not individually and independently carry out the corresponding enzymatic function. For example, acetyl-coa carboxylation is carried out by a multiprotein complex consisting of three subunits (one of which consists of two proteins); accA, accB, accC and accD. There were two sets of homologs in the genome annotated as biotin carboxylases accB (BC;usa300hou_rs08160, usa300hou_rs08560) and biotin carboxyl carrier protein accC (BCCP;usa300hou_rs08155, usa300hou_rs08555) sharing 35% and 47% sequence homology respectively. The usa300hou_rs08155 and usa300hou_rs8160 shared strong structural similarity to a biotin carboxylase in *S. aureus* and biotin carboxyl carrier protein in *E. coli* respectively. Incidentally, they were also contiguous. We could not find significant structural homology for the second set of genes. Additionally, one gene in the second set (usa300hou_rs08555) was observed to be non-essential while usa300hou_rs08155 was essential. Taken together, these results strongly suggest that usa300hou_rs08555 and usa300hou_rs08560 are functionally different from usa300hou_rs08155 and usa300hou_rs08160 respectively. Similarly, while two genes were annotated as serine-tRNA ligase, only one was found to be essential (usa300hou_rs00045 and not USA300HOU_RS08390). Again, upon examining the swissprot BLAST results, we found that the essential gene has high sequence homology with a serine-tRNA ligase experimentally characterized in *S. aureus* (16) while the other did not. As a result, we updated the gene reaction rule to only include usa300hou_rs00045.

Similarly, two genes were annotated as encoding uracil phosphoribosyltransferase (UPP;usa300hou_rs11405, usa300hou_rs06020) in the *S. aureus* genome. The usa300hou_rs11405 was observed to be non-essential and shared strong structural homology with the uracil phosphoribosyltransferase in *Bacillus caldolyticus* (17). The usa300hou_rs06020 was found to be essential and was annotated as pyrR, a bifunctional protein involved in regulation of pyrimidine biosynthetic genes but also known to have a weak uracil phosphoribosyltransferase activity in *Bacillus subtilis* (18). We hypothesize that pyrR is essential for its regulatory role as opposed to it UPP activity.

6. Mutants with partially affected growth corroborate the presence of functional isozymes in *S. aureus*:

Prediction results were variable for the 11 mutants whose growth was partially affected with respect to wild type. We found that 5 gene knockouts (*ald, ald2 pyc, argD* and *gtlA*) simulate unaffected growth. The mutant with a disrupted *gltA* (citrate synthase) had an intermediate growth phenotype but the model simulated full growth. The condensation of acetyl-coa and oxaloacetate can be catalyzed by two different isozymes; *gltA* or *sbnG*. SbnG is a second citrate synthase that shuttles citrate for staphyloferrin B biosynthesis (19). While both enzymes are wired to carry out a similar function, they are structurally distinct and participate in two different metabolic modules, which could explain the observed intermediate growth phenotype.

In another case, two genes - ald1 and ald2, annotated as alanine dehydrogenases and incorporated in an 'OR' rule were found to affect bacterial growth individually upon disruption. While the gene sequences only shared 40% percent sequence identity, the respective protein structures (obtained from homology modeling) aligned well, with an RMSD alignment of 0.94. The crystal structure used for homology modeling originated from a *Mycobacterium tuberculosis* alanine dehydrogenase. The two active site residues (His96 and Asp270) which were identified in the original structure (20), were found to be conserved in *ald1* and *ald2*. We further searched for homology across 46 sequences of alanine dehydrogenase spanning 5 organisms including *Bacillus halodurans*, *Thermus thermophilus*, *Archaeoglobus fulgidus, Mycobacterium tuberculosis* and *Phormidium lapideum* and found that while they may share the same function, the percent identity of the sequences ranged between 23.85% and 61.73%. Taken together, these results hint that both genes do function as alanine dehydrogenases and that they may have evolved to function in different metabolic modules.

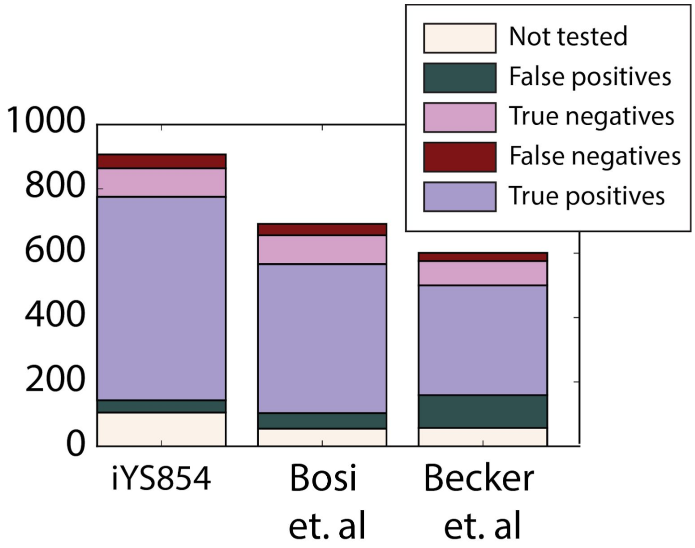

S1 Fig: Comparison of gene essentiality prediction accuracy across different *S. aureus* GEMs.

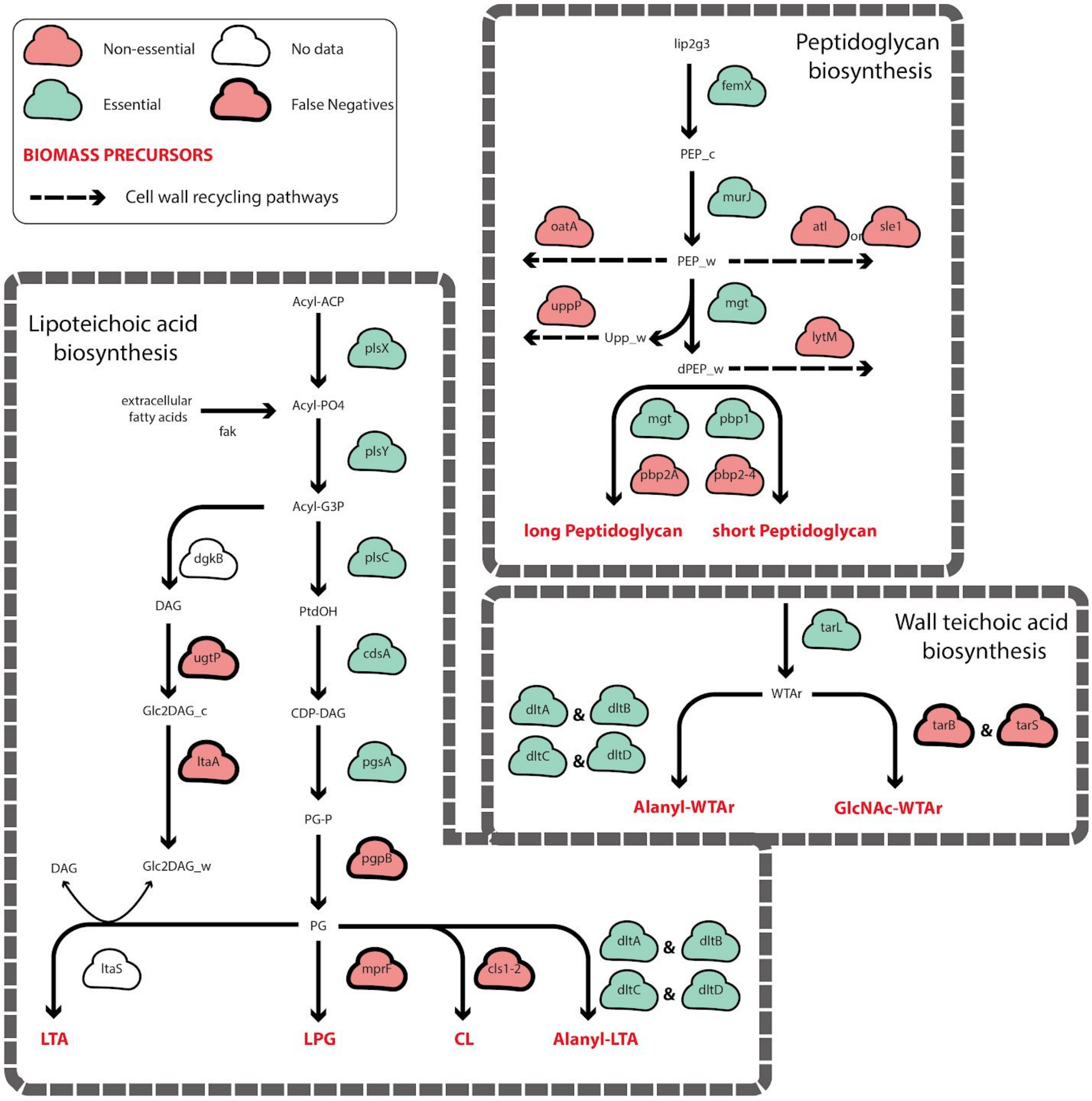

`

S2 Fig: Cell wall biosynthesis false negatives. We compared the GEM predictions for gene essentiality against the experimental observations of transposon mutant library. We found a total of 8 genes that were falsely predicted to be essential involved in the biosynthesis of cell wall components including lipoteichoic acid and wall teichoic acids.

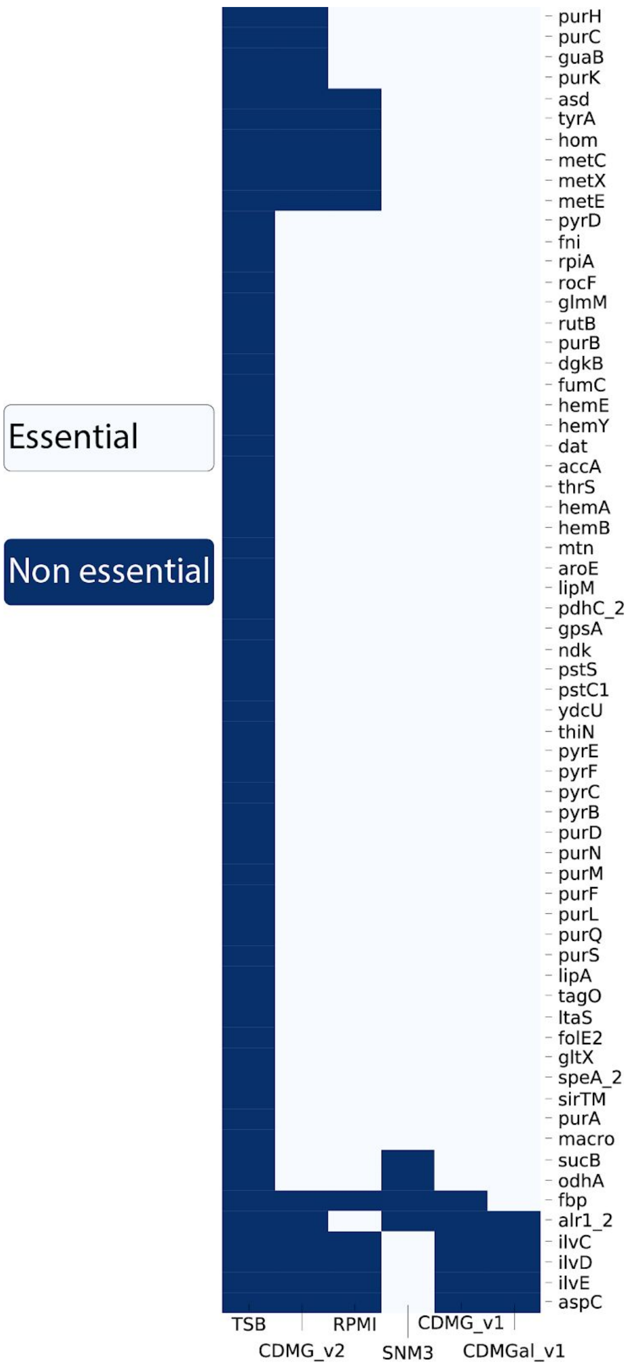

S3 Fig: Gene essentiality predictions for growth of *S. aureus* strains on multiple media types. We simulated the effect of a gene knockout on different chemically defined media *in silico*. The genes shown here are observed to be nonessential in TSB (*in vitro*) and the *in silico* model-driven predictions in 6 different media. For practicality, the genes that were falsely predicted to be essential in rich media (discussed in the main text) are not shown here. See table S3 for the full data-set.

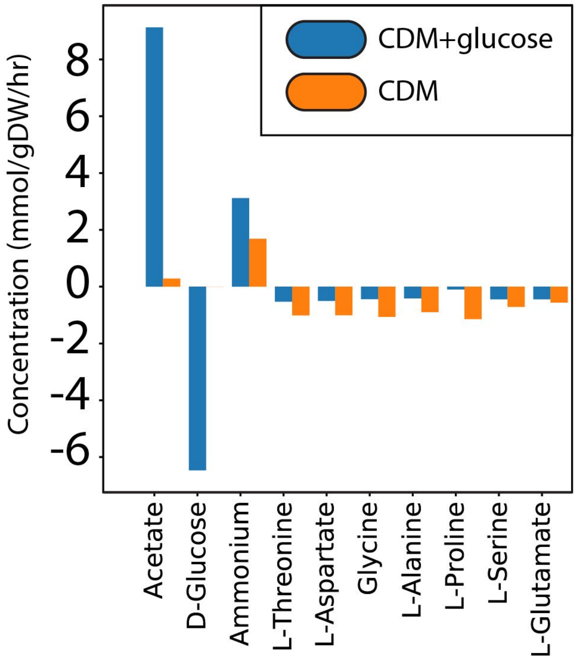

S4 Fig: Uptake rates for the top 10 extracellular metabolites calculated from the absolute quantitative exo-metabolomics measurements for growth of *S. aureus* strain LAC on chemically defined medium (CDM) and glucose + chemically defined medium (CDMG) (21). The full set of calculated uptake rates is available in table S4.

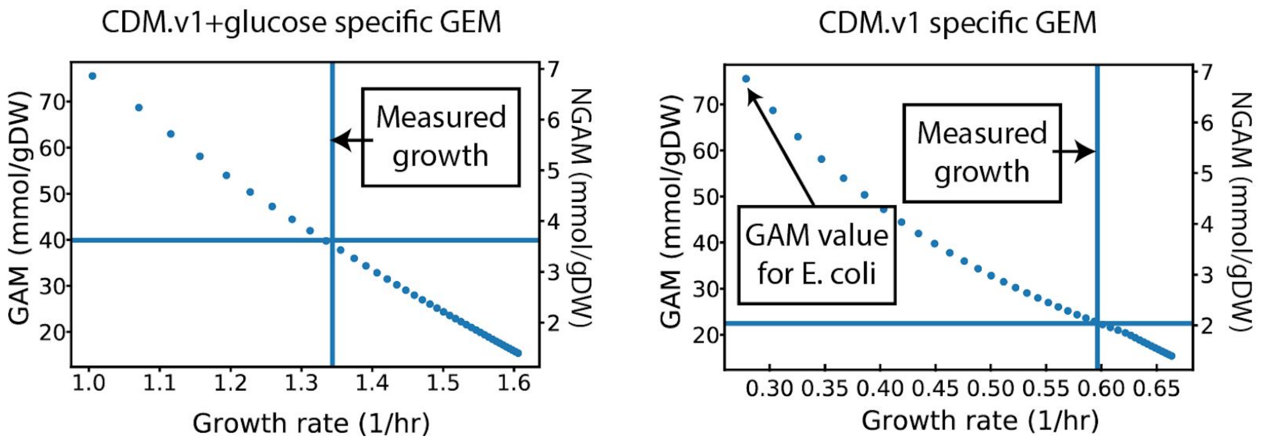

S5 Fig: GAM and NGAM calculation for *S. aureus*. For each condition-specific GEM, a GAM and NGAM value were calculated by taking a percentage of the *E. coli* GAM and NGAM and simulating for maximal growth. Maximal growth was simulated from 100% of the *E. coli* value down to 10% of the initial value. Experimentally observed growth is shown on the plot and its intersection with the GAM vs growth rate curve is used to find the corresponding GAM and NGAM values

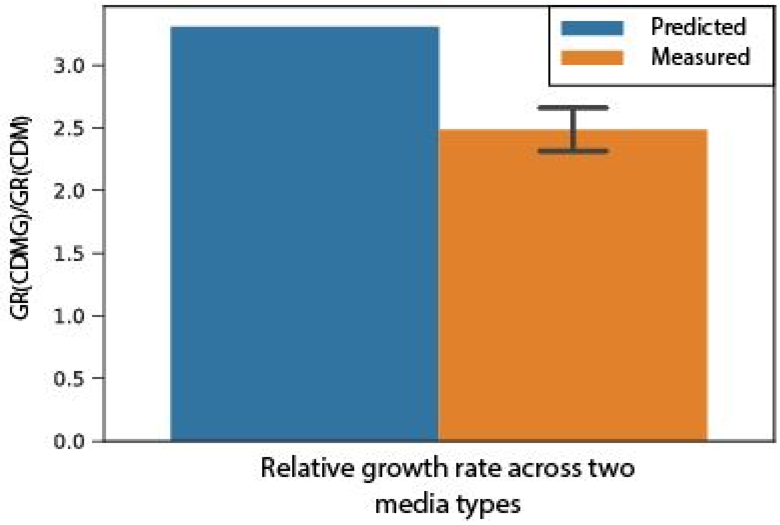

S6 Fig: Comparison of predicted and measured relative growth rate across the CDM versus CDM+glucose

1. Priyadarshi A, Kim EE, Hwang KY. Structural insights into Staphylococcus aureus enoyl-ACP reductase (FabI), in complex with NADP and triclosan. Proteins: Struct Funct Bioinf. 2010;78(2):480–6.

2. Schiebel J, Chang A, Lu H, Baxter MV, Tonge PJ, Kisker C. Staphylococcus aureus FabI: inhibition, substrate recognition, and potential implications for in vivo essentiality. Structure. 2012 May 9;20(5):802–13.

3. Scaletti ER, Luckner SR, Krause KL. Structural features and kinetic characterization of alanine racemase from Staphylococcus aureus (Mu50). Acta Crystallogr D Biol Crystallogr. 2012 Jan;68(Pt 1):82–92.

4. Hammer ND, Skaar EP. Molecular mechanisms of Staphylococcus aureus iron acquisition. Annu Rev Microbiol. 2011;65:129–47.

5. Bosi E, Monk JM, Aziz RK, Fondi M, Nizet V, Palsson BØ. Comparative genome-scale modelling of Staphylococcus aureus strains identifies strain-specific metabolic capabilities linked to pathogenicity. Proc Natl Acad Sci U S A. 2016 Jun 28;113(26):E3801–9.

6. Monk JM, Charusanti P, Aziz RK, Lerman JA, Premyodhin N, Orth JD, et al. Genome-scale metabolic reconstructions of multiple Escherichia coli strains highlight strain-specific adaptations to nutritional environments. Proc Natl Acad Sci U S A. 2013 Dec 10;110(50):20338–43.

7. Joshi GS, Spontak JS, Klapper DG, Richardson AR. Arginine catabolic mobile element encoded speG abrogates the unique hypersensitivity of Staphylococcus aureus to exogenous polyamines. Mol Microbiol. 2011 Oct;82(1):9–20.

8. Wielders CLC, Fluit AC, Brisse S, Verhoef J, Schmitz FJ. mecA gene is widely

disseminated in Staphylococcus aureus population. J Clin Microbiol. 2002 Nov;40(11):3970–5.

9. Pereira SFF, Henriques AO, Pinho MG, de Lencastre H, Tomasz A. Role of PBP1 in cell division of Staphylococcus aureus. J Bacteriol. 2007 May;189(9):3525–31.

10. Łeski TA, Tomasz A. Role of penicillin-binding protein 2 (PBP2) in the antibiotic susceptibility and cell wall cross-linking of Staphylococcus aureus: evidence for the cooperative functioning of PBP2, PBP4, and PBP2A. J Bacteriol. 2005 Mar;187(5):1815–24.

11. Thiele I, Palsson BØ. A protocol for generating a high-quality genome-scale metabolic reconstruction. Nat Protoc. 2010 Jan;5(1):93–121.

12. Neubauer H, Pantel I, Lindgren PE, Götz F. Characterization of the molybdate transport system ModABC of Staphylococcus carnosus. Arch Microbiol. 1999 Aug;172(2):109–15.

13. Broddrick JT, Rubin BE, Welkie DG, Du N, Mih N, Diamond S, et al. Unique attributes of cyanobacterial metabolism revealed by improved genome-scale metabolic modeling and essential gene analysis. Proc Natl Acad Sci U S A. 2016 Dec 20;113(51):E8344–53.

14. Hiramatsu T, Kodama K, Kuroda T, Mizushima T, Tsuchiya T. A putative multisubunit Na+/H+ antiporter from Staphylococcus aureus. J Bacteriol. 1998 Dec;180(24):6642–8.

15. Swartz TH, Ito M, Ohira T, Natsui S, Hicks DB, Krulwich TA. Catalytic properties of Staphylococcus aureus and Bacillus members of the secondary cation/proton antiporter-3 (Mrp) family are revealed by an optimized assay in an Escherichia coli host. J Bacteriol. 2007 Apr;189(8):3081–90.

16. Bausch N, Seignovert L, Beaulande M, Leberman R, Härtlein M. Analysis and overexpression in Escherichia coli of a staphylococcal gene encoding seryl-tRNA synthetase. Biochim Biophys Acta. 1998 Apr 29;1397(2):169–74.

17. Kadziola A, Neuhard J, Larsen S. Structure of product-bound Bacillus caldolyticus uracil phosphoribosyltransferase confirms ordered sequential substrate binding. Acta Crystallogr D Biol Crystallogr. 2002 Jun;58(Pt 6 Pt 2):936–45.

18. Grabner GK, Switzer RL. Kinetic studies of the uracil phosphoribosyltransferase reaction catalyzed by the Bacillus subtilis pyrimidine attenuation regulatory protein PyrR. J Biol Chem. 2003 Feb 28;278(9):6921–7.

19. Kobylarz MJ, Grigg JC, Sheldon JR, Heinrichs DE, Murphy MEP. SbnG, a citrate synthase in Staphylococcus aureus: a new fold on an old enzyme. J Biol Chem. 2014 Dec 5;289(49):33797–807.

20. Agren D, Schneider G. Ald ternary complex [Internet]. 2008. Available from: http://dx.doi.org/10.2210/pdb2vhx/pdb

21. Halsey CR, Lei S, Wax JK, Lehman MK, Nuxoll AS, Steinke L, et al. Amino Acid

Catabolism in Staphylococcus aureus and the Function of Carbon Catabolite Repression. MBio [Internet]. 2017 Feb 14;8(1). Available from: http://dx.doi.org/10.1128/mBio.01434-16
